# Extracellular 70 kDa heat shock protein in blood plasma binds insulin and modulates glycaemic control *in vivo*

**DOI:** 10.1016/j.cstres.2026.100180

**Published:** 2026-05-10

**Authors:** Mirna Stela Ludwig, Thiago Gomes Heck, Vânia Cibele Minguetti-Câmara, Helena Trevisan Schroeder, Carlos Henrique de Lemos Muller, Aline Bittencourt, Sofia Pizzato Scomazzon, Maciel Alencar Bruxel, Rossana Rosa Porto, Bolívar Bertoldo Bandeira, Victor de Souza Borges, Pauline Brendler Goettems-Fiorin, Fernanda Giesel Baldissera, Cinthia Maria Schöler, Patricia Martins Bock, Maria Inês Lavina Rodrigues, Patrícia Renck Nunes, Ana Lúcia Hoefel, Antônio Azambuja Miragem, Mauricio Krause, Marcelo Sartori Grunwald, Conrado Pedebos, Rodrigo Ligabue-Braun, José Cláudio Fonseca Moreira, Rui Curi, Roberto Barbosa Bazotte, Paulo Ivo Homem de Bittencourt

**Affiliations:** 1 Laboratory of Cellular Physiology, Department of Physiology, Institute of Basic Health Sciences, Federal University of Rio Grande do Sul, Porto Alegre, Porto Alegre, Rio Grande do Sul, Brazil; 2 Laboratory of Biological Assays and Post Graduate Program in Integral Health Care (PPGAIS-UNIJUÍ/UNICRUZ/URI), Regional University of Northwestern Rio Grande Do Sul State (UNIJUI) and Post Graduate Program in Mathematical and Computational Modeling (PPGMMC), UNIJUI, Ijuí, Rio Grande do Sul, Brazil; 3 Postgraduate Program in Pharmaceutical Sciences, State University of Maringá (UEM), Maringá, Paraná State, Brazil; 4 Centro Universitário de Maringá, Maringá, Paraná, Brazil; 5 Laboratory of Inflammation, Metabolism and Exercise Research (LAPIMEX), Department of Physiology, ICBS, UFRGS, Porto Alegre, Rio Grande do Sul, 90035-003, Brazil; 6 BioNTech SE, Mainz, Rheinland-Pfalz, Germany; 7 Western Sydney University School of Medicine, Sydney, Australia; 8 Faculdade da Serra Gaúcha, Caxias do Sul, Rio Grande do Sul, Brazil; 9 Federal Institute of Education Science and Technology Farroupilha, Santa Rosa, Rio Grande do Sul, Brazil; 10 Department of Biochemistry, Institute of Basic Health Sciences, Federal University of Rio Grande do Sul, Porto Alegre, Rio Grande do Sul, Brazil; 11 Department of Pharmacosciences, Federal University of Health Sciences of Porto Alegre (UFCSPA), Porto Alegre, Rio Grande do Sul, Brazil; 12 Instituto Butantan School, Butantan Institute, São Paulo, São Paulo, Brazil; 13 Interdisciplinary Post-Graduate Program in Health Sciences, Cruzeiro do Sul University, (UNICSUL), São Paulo, São Paulo, Brazil; 14 Universidade de Marília, Marília, SãoPaulo, Brazil; 15 Cruzeiro do Sul University, São Paulo, São Paulo, Brazil

**Keywords:** Glucose homeostasis, HSP70, Heat shock response, Chronic inflammatory diseases, Obesity, Insulin resistance

## Abstract

**Background & hypothesis:**

The 70 kDa heat shock protein family (HSP70) preserves the three-dimensional integrity of intracellular proteins, preventing the formation of cytotoxic aggregates that activate inflammatory pathways. Both constitutive and stress-inducible HSP70 isoforms uphold proteostasis during the heat shock response (HSR), an evolutionarily conserved, anti-inflammatory mechanism that restores cellular homeostasis following proteotoxic stress and metabolic disruption. Under conditions threatening homeostasis—such as heat shock (HS) and physical exertion, in which the sympathetic nervous system is strongly activated—HSP70 may be secreted into the extracellular space, where it functions as an immunomodulator and pro-inflammatory danger signal. Chronic inflammatory conditions, including obesity and type 2 diabetes, are characterized by persistently elevated levels of extracellular HSP70 in the bloodstream, which correlate with insulin resistance and β-cell dysfunction. Notably, glucose ingestion blocks exercise-induced HSP70 secretion, suggesting a previously unrecognized role of extracellular HSP70 in modulating glycaemia via insulin binding.

**Results:**

Here we show a novel counterregulatory mechanism in which plasma HSP70 binds insulin with high affinity (K_d_ ∼3 pM), impairing glucose uptake in insulin-dependent tissues without affecting receptor signaling. In fasted rats subjected to HS, elevated plasma HSP70 raises glycaemia by ∼3 mM during glucose tolerance tests (at 30 min), enhancing glucose availability for non-insulin-dependent tissues. HS-induced glucose intolerance peaks 12 h post-HS in an HSP70-interacting protein (HIP)-dependent manner. However, HS enhances the insulinogenic index (IGI) and insulin sensitivity, peaking at 24 h.

**Conclusion:**

This observation challenges the paradigm that HSP70 functions to chaperone proteins solely intracellularly, revealing its role in extracellular glycaemic regulation by HIP-assisted protein-protein interactions in blood plasma, thus offering a novel clinical viewpoint in glycaemic management.

**Translational perspectives:**

These findings suggest that humanized anti-HSP70 monoclonal antibodies could mitigate insulin sequestration and offer a novel therapeutic strategy to restore insulin sensitivity, thereby improving metabolic outcomes in conditions with elevated plasma HSP70, such as type 2 diabetes, obesity, non-alcoholic fatty liver disease, and cardiovascular disease.

## Highlights

• Plasma HSP70 binds insulin with high affinity, impairing glucose uptake without affecting insulin signalling.
• Heat stress induces transient glucose intolerance via an HSP70/HIP-dependent glucose redistribution.
• Plasma HSP70 emerges as an extracellular regulator of glycaemia, offering a novel clinical viewpoint.
• *Targeting extracellular HSP70 with humanized monoclonal antibodies may restore insulin sensitivity in inflammatory metabolic diseases*.

## Graphical Abstract

**Figure:**
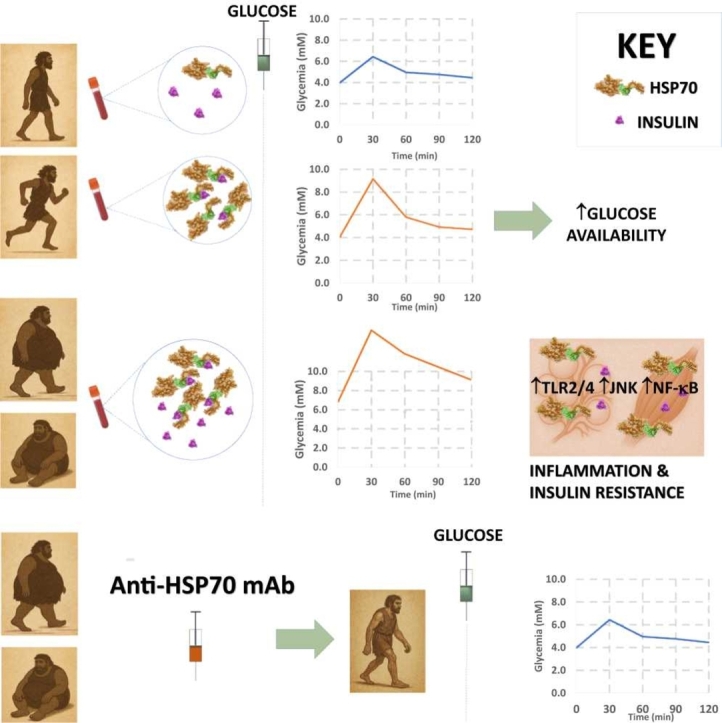
During stress-associated sympathetic activation (e.g., prolonged fasting, exercise, heat stress), HSP70 is released into the bloodstream and binds insulin in the presence of HSP70-interacting protein (HIP), redirecting glucose toward insulin-independent tissues. Conversely, in obesity-related inflammatory diseases, elevated plasma HSP70 triggers inflammation via Toll-like receptors and sequesters insulin, driving compensatory hyperinsulinaemia and worsening insulin resistance. In such cases, treatment with humanized anti-HSP70 monoclonal antibodies may alleviate inflammation and improve inflammation-related obesity and insulin sensitivity.

## Background

Various physiologically stressful conditions, including heat shock (HS) and physical exercise, elevate the production of the 70 kDa family of heat shock proteins (HSP70), both intra and extracellularly.^1^ This rise in circulating HSP70 precedes any increase in HSP70 gene or protein expression in contracting muscle, suggesting its release from other tissues or organs. Hepatosplanchnic tissues have been identified as a primary source, with HSP70 levels remaining elevated above baseline even 24 h after exercise, correlating with increased HSP70 gene expression in active muscles.^2^ Peripheral blood leukocytes also contribute to HSP70 production in plasma under stress,^3^, ^4^ with lymphocytes accounting for nearly all peripheral blood leukocytes-derived HSP70.^3^

HSP70 export to the extracellular space occurs in various cell types^5^, ^6^ and is independent of passive release due to cell death,^3^ relying instead on exosome trafficking.^7^ Beyond exercise and heat treatment, other stressors—such as trauma, severe infection, and psychological stress—also elevate HSP70 plasma levels.^8^ This release is mediated by the α₁-adrenoreceptor signaling pathway and is independent of β-adrenoreceptors, glucocorticoids, and ACTH.^9^ Notably, glucose ingestion significantly reduces the exercise-induced release of HSP70 into the bloodstream.^10^

Acutely, during hypoglycemic challenges induced by insulin administration, circulating HSP70 levels rise in parallel with pro-inflammatory cytokine release.^11^ Chronically, subjects with type 2 diabetes mellitus (T2DM), who often present hyperinsulinemia together with peripheral insulin resistance, also show elevated HSP70 and pro-inflammatory markers, independent of glycemic levels.^12^ To date, therefore, extracellular HSP70 has been primarily associated with pro-inflammatory responses—unlike the anti-inflammatory role of intracellular HSP70^4^, ^13^—following both acute and chronic metabolic stress, acting as a signaling molecule (chaperokine) rather than as a direct regulator of glucose metabolism. This is consistent with the view that insulin resistance, frequently coexisting with hyperinsulinemia in T2DM, is closely linked to inflammation and oxidative stress.

Altogether, these findings led us to hypothesize that, under predominantly sympathetic stimuli (e.g., prolonged fasting, exercise, HS, diabetes), HSP70 is released into circulation as an extracellular chaperone that could bind insulin, attenuating its effects to prevent hypoglycemia during acute stress. This hypothesis is reinforced by the fact that the heat shock response (HSR)—the biochemical pathway from HuR (ELAV-1) to heat shock factor-1 (HSF1)-driven HSP70 production and release—is closely linked to energy metabolism, particularly glucose metabolism, and is regulated by 5’-adenosine monophosphate-activated protein kinase (AMPK), the master energy sensor in all cells.^14^, ^15^ Indeed, the HSP70 molecule contains a hexokinase domain, which originated 3.6 billion years ago, suggesting parallel evolution between HSP chaperones and sugar kinases.^14^

Skeletal muscle takes up glucose independently of insulin during exercise via an AMPK-dependent mechanism,^16^, ^17^ whereas insulin (t₁/₂ = 4-6 min) persists in circulation for over an hour.^18^, ^19^ In a fed state, its concentration (maximum ∼800 pM) may require more than half an hour to return to fasting levels (18-90 pM). Moreover, even after its removal from circulation, insulin’s intracellular signaling and hypoglycemic effects may persist substantially longer. During this window, any sympathetic nervous system-centered stress response (e.g., heavy exercise, fight-or-flight situation) could critically decrease glucose availability, posing a fatal risk. Although counter-regulatory mechanisms—including catecholamine release, glucagon secretion, and other hormonal responses—act to preserve glycemic homeostasis, their effects may not be sufficiently rapid to fully offset ongoing insulin action, particularly in the presence of sustained insulin-independent glucose uptake by skeletal muscle. Thus, we hypothesized that, under sympathetic stimuli, plasma HSP70 could act as an extracellular modulator of insulin availability, attenuating its effects and thereby contributing to the prevention of stress-induced hypoglycemia.

## Results

### HSP70 binds to and sequesters insulin *in silico*, *in vitro* and *in vivo*

**Fig. 1:**
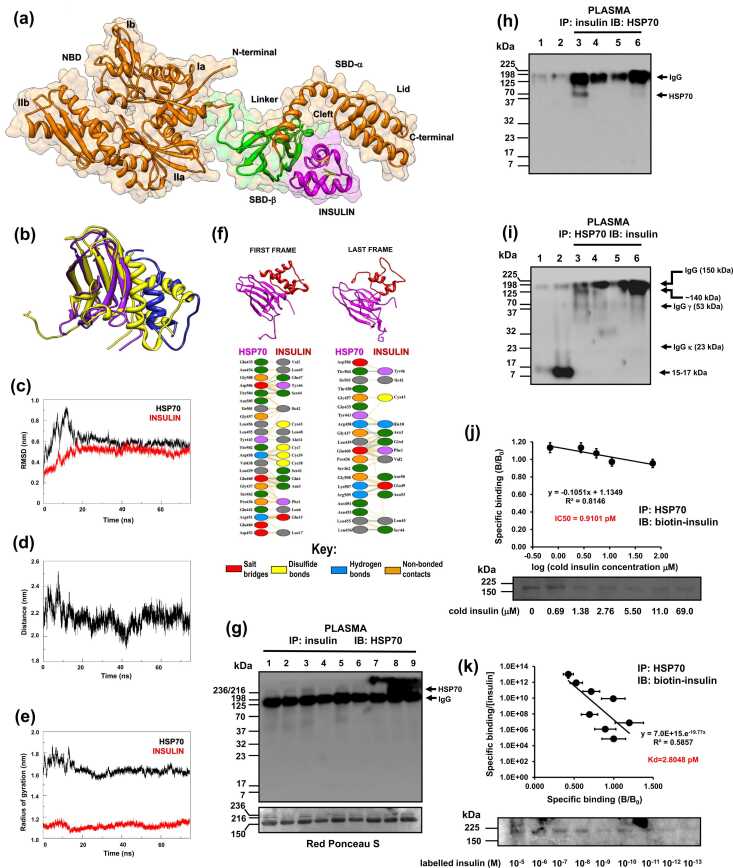
*HSP70 binds to and sequesters insulin in silico, in vitro and in vivo.***(a)** The molecular model of HSP70 (HSPA1A) was generated with *MODELLER v9.8.* The best model out of fifty was chosen based on the parameters given by the server PDBsum (http://www.ebi.ac.uk/pdbsum/). Our model comprises the 45 kDa nucleotide-binding domain [(NBD), including subdomains Ia (residues 1-39 and 115-188), Ib(39-115), IIa (188-228 and 306-385)], the 30 kDa substrate-binding domain (SBD, showing SBD-α and SBD-β domains, the cleft on SBD, and the lid near the C-terminal), and the C-terminal domain (Gln603) of human HSP70 (orange). The linker region (green) and insulin (magenta) are also shown. **(b)** The trajectory of the molecular dynamics (MD) is represented by the superimposition of the initial frame of the docking run between the HSP70 SBD and insulin, which are shown in yellow. The final frame structure of HSP70 SBD is given in magenta, and that of insulin in blue. Different rotational perspectives of part (a) are depicted in Supplementary Figure 1a while the trajectory of the MD run of part (b) is provided under different rotational perspectives in Supplementary Figure 1b. **(c)** Time-dependence of structural parameters from molecular dynamics (MD) simulations showing atom-positional root mean square deviation (RMSD) of all atoms of the docking solution between HSP70 PBD and insulin. **(d)** Distance between the center of mass of the two components of the system. **(e)** The radius of gyration of the components of the system separately. **(f) Upper part**: Extracts from the protein-protein interaction diagrams in PDBsum for PDB resulting from the initial and final frames of the MD docking run for the solution between HSP70 SBD (magenta) and insulin (red). **Middle part**: Thumbnail image of the 3D structural docking solution showing the initial and final frame models. Details of the individual residue-residue interactions across this interface from the initial and final frames of the MD run of the docking solution. The nature of such interactions is represented in the color key. **(g)** Rat plasma samples were immunoprecipitated (IP) for insulin and immunoblotted (IB) for HSP70 under the following experimental conditions (lanes): **1)** fed, **2)** 15 h fasted, **3)** 15 h fasted + Detemir insulin (killed 4 h after insulin), **4)** 15 h fasted + Detemir insulin (killed 10 h after insulin), **5)** fed, **6)** 6 h fasted, **7)** 6 h fasted + Lispro insulin (killed 15 min after insulin), **8)** 6 h fasted + Lispro insulin (killed 60 min after insulin, **9)** 6 h fasted + Lispro insulin (killed 180 min after insulin). To verify the specificity of insulin-HSP70 interactions, samples underwent IP for insulin followed by IB to HSP70 **(h)** or IP for HSP70 and IB to insulin **(i),** after native electrophoresis. The following conditions were tested (lanes): **1)** 139 pM HSP70 in sample buffer, **2)** 139 pM insulin in sample buffer; **IP: 3)** 139 pM HSP70 in whole plasma from 12 h fasted rats, **4)** 139 pM HSP70 + 139 pM insulin in dialyzed rat serum, **5)** 139 pM insulin in dialyzed rat serum, **6)** negative control with whole plasma and IP with non-immune mouse IgG from normal mouse serum. **(j)** Eadie-Hofstee plot of competition analysis: 1.38 μM HSP70 was incubated in the presence of 1.38 μM biotin-insulin and increasing concentrations of cold insulin in dialyzed FBS for 4 h at 37 °C. IP for HSP70 followed by streptavidin IB for biotin-insulin. IC50 = half-maximal inhibitory concentration. Data are the means ± s.d. (*n* = 4). **(k)** Scatchard plot for K_d_ determination: 1.38 μM HSP70 was incubated in the presence of increasing concentrations of biotin-insulin (from 10^−13^ to 10^−5^ M) in dialyzed FBS for 4 h at 37 °C. IP for HSP70 followed by streptavidin IB for biotin-insulin. K_d_ = dissociation constant. Data are the means ± s.d. (*n* = 4).

To test our hypothesis, we firstly conducted molecular dynamics (MD) and docking experiments to determine whether HSP70 interacts with insulin. We refined the docking solution between HSP70 (HSPA1A)’s substrate-binding domain (SBD) and insulin using MD (Figure 1a, Supplementary Figure 1a). The results revealed that insulin binds precisely within HSP70’s SBD cleft.

The MD trajectory is illustrated by superimposing the initial docking frame (yellow) with the final MD frame (HSP70 SBD in magenta, insulin in blue) (Figure 1b). Docking analysis identified key interaction regions: insulin’s Phe1–Leu17 and Cys39–Leu45, and HSP70’s Leu403–Ala412, Gly437–Glu444, and Thr450–Ser462. Stability was assessed via the root mean square deviation (RMSD), which showed system stabilization within 20 ns of simulation (Figure 1c). The center-of-mass distance between the proteins remained constant (Figure 1d), supported by the radius of gyration analysis indicating structural compaction (Figure 1e). Figure 1f (upper insets, Appendix A, Appendix A) shows the superimposed initial and final MD frames. Protein-protein interaction analysis (PDBsum) of the first MD frame revealed a contact surface of 1036 Å² for HSP70’s SBD (23 interacting residues) and 1091 Å² for insulin (19 interacting residues), primarily mediated by 102 non-bonded contacts and 8 hydrogen bonds (Figure 1f, middle panel). By the final MD frame, the contact surfaces reduced to 870 Å² (20 residues) for HSP70 and 989 Å² (13 residues) for insulin, with 96 non-bonded contacts and 12 hydrogen bonds. These results suggest a stable interaction between HSP70’s SBD and insulin, as confirmed by molecular docking and refined by MD. The system remained highly stable throughout 60 ns of simulation, with significant protein-protein interface interactions. A second set of MD experiments using *Rattus norvegicus* hspa1a yielded identical results (please, see below).

To investigate whether fasting or insulin-induced hypoglycemia influences HSP70-insulin binding in circulation, we immunoprecipitated plasma samples from fed, fasted, and fasted insulin-treated rats using an anti-insulin antibody, followed by immunoblotting for HSP70 (hspa1a+hspa8). Figure 1g (see legend) shows that insulin immunoprecipitation co-purified HSP70, forming two high-molecular-weight bands (216 and 236 kDa). Co-immunoprecipitation was more pronounced in fasted insulin-treated rats than in fed or fasted-only controls. Two insulin types were tested: Humalog Lispro (fast-acting) and Detemir (myristoyl-insulin, slower-acting). High-sensitivity quantification (ELISA) of inducible HSP70 (hspa1a+hspa1b) revealed an insulin-induced hypoglycemia-dependent increase in HSP70 from 0.012 to 0.176 ng/mL in immunoprecipitated plasma. This result corroborates our previous findings that low glucose levels (down to 1.56 ± 0.14 mM), rather than insulin, trigger a sharp increase in HSP70 plasma concentrations and hepatic expression, an effect linked to elevated IL-6 levels but not IL-10 or TNF-α.^11^

To further examine HSP70-insulin binding, equimolar amounts of mouse hspa1a (139 pM, equivalent to 10 ng/mL in plasma) and Humalog Lispro insulin were incubated for 90 min at 37 °C in Hanks’ balanced salt solution (HBSS). Immunoprecipitation using either anti-insulin or anti-HSP70 antibodies followed by native discontinuous gradient electrophoresis showed no co-immunoprecipitation, suggesting that an additional plasma component is required for binding. Since HBSS incubation did not replicate the *in vivo* interaction, we repeated the experiment in dialyzed serum or whole plasma from 12 h-fasted rats under the same conditions. Immunoprecipitation for insulin then co-purified HSP70 (Figure 1h, lane 3), while immunoprecipitation for HSP70 co-purified insulin (Figure 1**i**, lanes 2, 3, 4), confirming plasma-dependent binding. Control experiments (lanes 5 and 6 in Figure 1h and i) ruled out antibody cross-reactivity and nonspecific immunoprecipitation.

For competition and dissociation constant (K_d_) calculations, samples were incubated in dialyzed foetal bovine serum (FBS) or 12 h-fasted rat serum with increasing concentrations of Humalog Lispro insulin to be immunoprecipitated and immunoblotted (Figure 1j). Binding assays with 1.38 μM biotinylated insulin and 1.38 μM HSP70 in dialyzed serum revealed an IC50 of 0.91 pM, as determined by Eadie-Hofstee analysis (Figure 1j), and a K_d_ of 2.80 pM, visualized in the Scatchard plot (Figure 1k).

### HS promotes glucose intolerance 12 h after heat treatment while improving insulin sensitivity up to 24 h after HS

Given that HSP70 binds to insulin, a key question arises: what is the physiological significance of this interaction? Since HSP70 and its accompanying HSR were evolutionarily selected for key defensive functions in stress responses, this binding likely serves a vital purpose. One possibility is that HSP70 temporarily reduces glucose tolerance during stress by sequestering insulin, mitigating the risk of severe hypoglycemia. Since insulin persists in circulation for over an hour after secretion,^18^, ^19^ prolonged activity in stress conditions could pose a significant threat to glycemic stability.

In order to test this hypothesis, we subjected fasted and fed rats to HS stress (HS; 42 °C for 15 min) and performed intraperitoneal glucose (ipGTT) and insulin (ipITT) tolerance tests at various time points. HS triggers a hyperadrenergic state to sustain cardiac output,^20^ as increased skin blood flow and sweating necessitate sympathetic adjustments to maintain blood pressure and brain perfusion. This response stimulates adrenoreceptors in metabolic tissues—skeletal muscle, adipose tissue, liver, and pancreas^21^—and enhances HSP70 expression and secretion into the extracellular space and then into circulation. This eventually elevates plasma HSP70 levels. We hypothesized that this rise in circulating HSP70 would sequester insulin, impairing its action and thus reducing glucose tolerance.

**Fig. 2:**
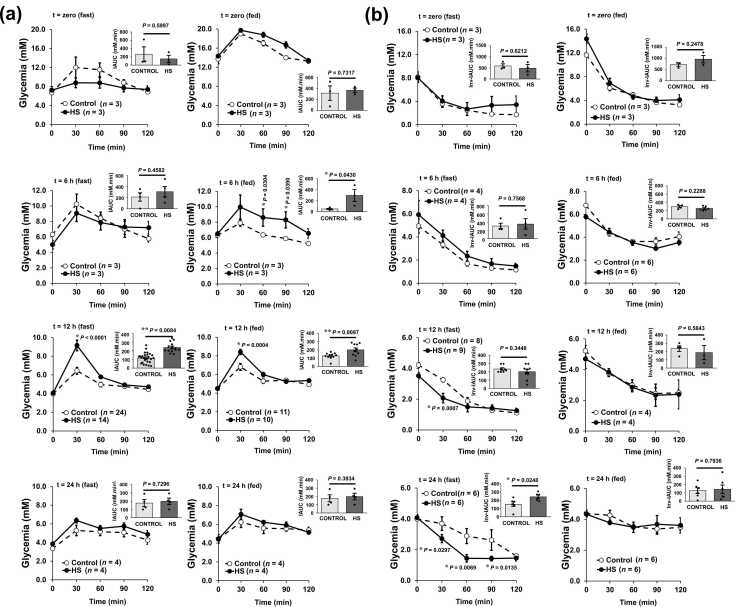
*HS promotes glucose intolerance 12 h after heat treatment while improving insulin sensitivity up to 24 h after HS treatment.***(a)** ipGTT was carried out just after HS (time zero) or after 6, 12, or 24 h after heat treatment in 12 h fasted (left) or fed (right) rats. **(b)** ipITT was conducted just after (time zero) or after 6, 12, or 24 h after heat treatment in 12 h fasted (left) or fed (right) rats. Differences in glycemic curves were assessed by 2-way RM ANOVA followed by Tukey’s multiple comparison testing, whereas iAUC of ipGTT and inv-iAUC of ipITT were evaluated by unpaired two-tailed Student’s *t*-test, as described in the Methods. Individual adjusted *P*-values are given when appropriate. Data are the means ± s.d. (sample size is indicated for each group). The complete set of experimental groups in ipITT is shown in Supplementary Figure 2 while plasma contents of HSP72, HSP73, total HSP70, insulin alongside HOMA-IR, QUICKI, and FFA plasma levels during ipGTT are depicted in Supplementary Figure 3. Complete data on plasma HSP72, HSP73, total HSP70, and insulin concentrations during the 30 min point of ipITT are given in Supplementary Figure 4.

The results confirmed the prediction: HS significantly increased the glycemic peak (30 min) during ipGTT by 2.68 ± 0.57 mM (*P*< .0001) in fasted and by 1.60 ± 0.51 mM (*P*< .0004) in fed animals, with a maximal effect at 12 h post-HS (Figure 2a). This represents a rise in iAUC of 70 % (*P* = .0084) in fasted and 57 % (*P* = .0097) in fed rats. However, in ipITT, insulin sensitivity improved from 12 to 24 h after HS (Figure 2b**,** Supplementary Figure 2), increasing inv-iAUC by 57 % (*P* = .0248) 24 h after HS. ELISA measurements of inducible HSP72 (hspa1a and hspa1b), constitutive HSP73 (hspa8), and total HSP70 (HSP72+HSP73) showed peak plasma concentrations between 12 and 24 h post-HS in fasted animals in ipGTT (Supplementary Figure 3a) and 24 h after HS in ipITT (Supplementary Figure 4).

Remarkably, glucose overload in HS-treated animals resulted in a striking 166 % increase in insulinemia (*P* = 0.0124), accompanied by a moderate yet consistent rise in the insulinogenic index [median (95 % CI): from 1.23 (1.00-1.46) to 1.56 (1.53-1.59), *P* < .001]. Hence, HS enhances the insulinogenic response to glucose, initially impairing glucose tolerance but subsequently improving insulin sensitivity. This is reflected in an improved HOMA-IR index 24 h post-HS (Supplementary Figure 3b), despite no observed change in the QUICK index (Supplementary Figure 3c). To exclude the possibility that HS-induced plasma free fatty acid (FFA) elevation might impair glucose utilization, we measured FFA levels, which remained stable over 24 h and even declined immediately post-HS (Supplementary Figure 3d). Lactate levels were unaffected by HS and remained unchanged (3.3 ± 0.3 mM) after insulin injections. The same was observed for plasma triacylglycerol (triglyceride) levels, which remained unaltered from basal 127.1 ± 20.6 to 103.7 ± 13.3 mg/dL (mean ± s.d.). Furthermore, the surge of insulin during ipGTT 12 h after HS (Supplementary Figure 3a) refutes the possibility that HS-elicited hyperglycemia could be due to low insulin levels.

### Hyperglycaemic response to HS is abolished by anti-HSP70 mAb and mimicked by the intravenous HSP70 plus HIP injection

**Fig. 3:**
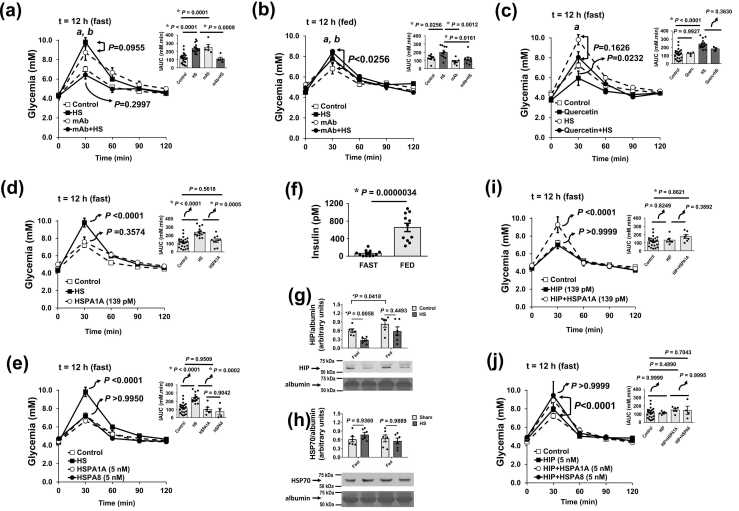
*HS hyperglycemic effects during ipGTT are not mimicked by HSP70 i.v. injection but is completely re-established by the concomitant injection of HSP70 plus HSP70-interacting protein (HIP).* Administration of anti-HSP70 mAb (3A3, which recognizes hspa1a, hspa1b, and hspa8 isoforms of HSP70) also completely abolished HS effects. The rats were 12 h fasted **(a)** or maintained fed **(b)** to be submitted to a HS session (and/or mAb injection) and underwent an ipGTT 12 h after heat (or mAb) treatment. **(c)** In order to evaluate whether hyperglycemic effects of HS are dependent of the *de novo* synthesis of HSP70 molecules, 12 h fasted animals were treated intragastrically (gavage) with 200 mg/kg of quercetin dihydrate (in water) 1 h before HS treatment, in order to block HSP70 biosynthesis. Twelve hours after HS (or quercetin + HS), the rats underwent a conventional ipGTT. To assess whether HSP70 alone could reproduce HS hyperglycemic effects during ipGTT, the animals were submitted to HS or injected (i.v.) with HSP70 in amounts that were estimated to reach d insulin during anti-HSP70 **(d)** 139 pM hspa1a isoform of HSP70 (equivalent to 10 ng/mL in plasma) and **(e)** with either 5 nM hspa1a or 5 nM hspa8 isoforms of HSP70. These latter concentrations equal 10 times the maximal insulin concentrations observed in fed animals **(f)**. Plasma concentrations of HSP70 (HSP72 = hspa1a and hspa1b; HSP73 = hspa8), total HSP70, and insulin during anti-HSP70 mAb, quercetin, and hspa1a injections are depicted in Supplementary Figure 5. As HSP70 injection alone did not reproduce HS hyperglycemic effects during ipGTT, we investigated whether some other partner chaperone or co-chaperone could be present in the plasma of the animals 12 h after HS sessions that could mimic HS effects. We tested (immunoblotting) DnaJA1 (HSP40), HSP20 (hspb6), BAG-1, HspBP1, and HSP70-interacting protein (HIP), as these proteins are known to mediate the interactions of ATP-dependent HSPs and client proteins. The results showed only the presence of HIP **(g)** and HSP70 **(h)** in the plasma of both 12 h fasted and fed rats. Therefore, we treated 12 h fasted animals with 139 pM hspa1a + 139 pM HIP **(i)** or with 5 nM hspa1a (or 5 nM hspa8) + 5 nM HIP **(j)** 12 h before the ipGTT tests. Glycemic curves were analyzed by 2-way RM ANOVA followed by Tukey’s multiple comparison testing. Differences between iAUC were assessed by 1-way ANOVA followed by Tukey’s multiple comparison testing. All the data are expressed as the means ± s.d. Sample size was: **(a)***n* = 24 for the controls, *n* = 14 for HS, *n* = 5 for mAb and *n* = 5 for mAb + HS; **(b)***n* = 11 for the controls, *n* = 10 for HS, *n* = 7 for mAb and *n* = 9 for mAb + HS; **(c)***n* = 24 for the controls, *n* = 14 for HS, *n* = 4 for quercetin and *n* = 4 for quercetin + HS; **(d)***n* = 24 for the controls, *n* = 14 for HS, *n* = 10 for 139 pM hspa1a injection; **(e)***n* = 24 for the controls, *n* = 14 for HS, *n* = 6 for 5 nM hspa1a injection, and *n =* 4 for 5 nM hspa8 injection; **(f)***n* = for fasted and *n* = 11 for fed animals; **(g)** and **(h)***n* = 6 for all the groups; **(i)***n* = 24 for the controls, *n* = 6 for 139 pM HIP, *n* = 7 for 139 pM HIP + 139 pM hspa1a; **(j)***n* = 24 for the controls, *n* = 6 for 5 nM HIP, *n* = 6 for 5 nM HIP + 5 nM hspa1a, *n* = 4 for 5 nM HIP + 5 nM hspa8.

If HSP70 binding to insulin induces glucose intolerance 12 h after HS, then administering anti-HSP70 monoclonal antibodies (mAb) 12 h before ipGTT should abolish this hyperglycemic effect. As expected, mAb against HSP70 (targeting both hspa1a and hspa8) completely abolished (*P*= .0003) the 30 min hyperglycemic peak in fasted rats (Figure 3a) and, to a lesser extent, in fed animals (Figure 3b).

However, does this effect rely on *de novo* HSP70 synthesis, or is it driven by the release of preassembled exosome vesicles? To address this, we pretreated fasted animals 2 h before HS with quercetin, a potent inhibitor of HSP70 synthesis at both transcriptional and post-transcriptional levels.^22^ Quercetin treatment did not affect the hyperglycemic response to HS 12 h later (Figure 3c); HSP70 and insulin levels during mAb, quercetin, and hspa1a injections are presented in Supplementary Figure. 5.

To determine whether HSP70 alone could reproduce the hyperglycemic effects of HS during ipGTT, animals underwent HS or received intravenous injections of HSP70 at estimated plasma concentrations of 139 pM hspa1a (equivalent to 10 ng/mL during ipGTT; Supplementary Figure 3) or 5 nM hspa1a/hspa8 – tenfold the maximal insulin concentration observed in fed animals (Figure 3**f**). However, neither hspa1a nor hspa8 injections at either concentration (139 pM or 5 nM) replicated HS-induced hyperglycemia (Figure 3d, e). Therefore, to explore whether other co-chaperones or auxiliary proteins contribute to HS-induced glycemic changes, we screened rat plasma (via Western blotting) for HspBP1, HSP70-interacting protein (HIP), HSP40 (DnaJA1), HSP20 (hspb6), and BAG1. Only HIP was detected (Figure 3g), with plasma HIP levels reduced by 50 % in fasted rats following HS. While we also measured HSP70, immunoblotting lacked the sensitivity to detect plasma HSP70 changes (Figure 3h).

Intracellularly, HIP collaborates with HSP70 in protein folding and aggregation prevention. HSP70 binds non-native protein substrates in an ATP-dependent cycle regulated by J-domain proteins and nucleotide exchange factors. HIP is believed to prolong substrate retention by delaying ADP dissociation from HSP70.^23^ We therefore treated fasted rats with HIP alone or in combination with HSP70 (hspa1a or hspa8). HIP alone did not induce hyperglycemia at either tested dose (139 pM or 5 nM) during ipGTT. However, co-injection of HIP with HSP70 reproduced the hyperglycemic response observed during HS (Figure 3i, j), with a peak increase in glycaemia (2.05 ± 0.93 mM at 30 min) relative to controls, reaching maximal levels even at the lower dose (*P* < .0001). Notably, this provides the first evidence that HIP can act extracellularly to support HSP70 function.

**Fig. 4:**
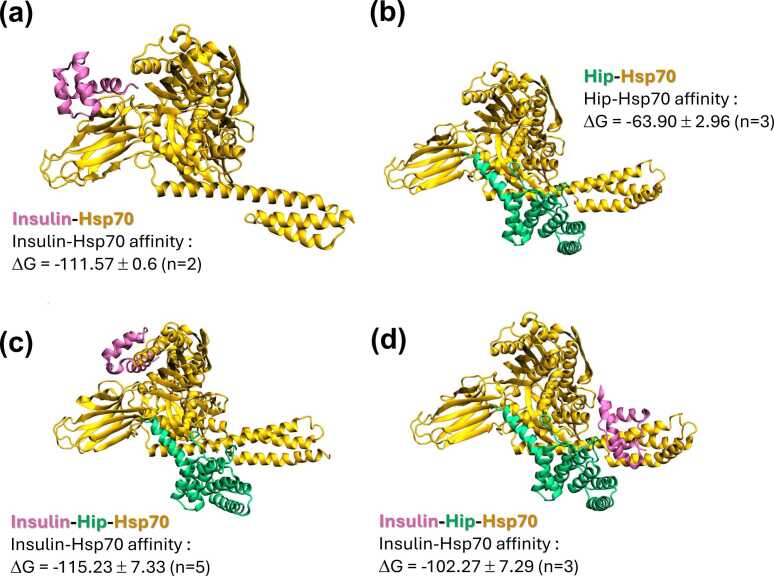
*Highest-ranked solutions for rat HSP70-HIP-insulin interactions as obtained by LightDock docking simulations.***(a)** Insulin (pink cartoon) binding to rat HSP70 (orange) at **site 1**, near the SBD-β domain. **(b)** Rat HIP (green) binding to rat HSP70 (orange) near the dimerization interface. **(c)** Tertiary complex formed by rat HIP (green), insulin (pink), and rat HSP70 (orange) showing insulin binding to **site 2**, near the HIP protein and the dimerization interface of HSP70. **(d)** Second-highest ranked solution of a tertiary complex formed by rat Hip (green), insulin (pink), and rat HSP70 (orange) showing insulin binding to **site 1**, far from HIP and near the SBD-β domain. For protein interactions’ affinities, ΔG values are given in Rosetta Energy Units (REU, ∼ kcal/mol).

These findings prompted new molecular modeling analyses to investigate potential HIP-mediated HSP70–insulin interactions using rat (*Rattus norvegicus*) proteins. The second round of *in silico* experiments, incorporating rat hspa1a, HIP, and insulin, is summarized in Figure 4. Briefly, binding energies (Rosetta Energy Units (REU)^24^) for both insulin and HIP78–247 increased when bound together to hspa1a, suggesting cooperativity. Insulin binding to hspa1a alone occurred primarily at the interface between the SBD-β barrel and NBD (Figure 1**a**), with hydrogen bonds forming between insulin and hspa1a residues 416, 417, 419, and 448. Additional contacts involved residues 221, 324, 394, 400, 405, 409, 412, 413, and 415.

When MD simulations included insulin bound to the hspa1a-HIP complex, two binding sites emerged: one resembling the primary site on hspa1a alone (site 1) and another at the SBD-α region (site 2), involving key residues 27, 32, 129, 134, 537, 541, 545, 593, 596, 608, 611, 612, and 614. This region is implicated in the dimerization of the HSP110:HSC70 nucleotide exchange complex (PDB ID 3C7N; Figure 1).^25^ Binding at site 2 may interfere with oligomerization, potentially preventing higher-order complex formation. Additionally, insulin at site 2 interacts directly with HIP, with HIP residues 16-20, 54, and 81 playing a role.

The average ΔG for hspa1a–insulin increased from −42.68 to −115.23 REU (site 1; Figure 4c) and to −102.99 REU (site 2; Figure 4d) in the presence of HIP. Similarly, HIP–hspa1a ΔG increased from −63.9 to −102.27 REU (site 1; Figure 4b) and to −111.57 REU (site 2; Figure 4**a**) when insulin was present. These findings likely explain why HSP70 induces glucose intolerance in the presence of HIP during ipGTT (Figure 3i,j) when glucose-induced insulin release is heightened (Supplementary Figure 5).

### HS and HSP70 injection impair glucose uptake and utilization in insulin-dependent tissues without affecting insulin receptor signaling

**Fig. 5:**
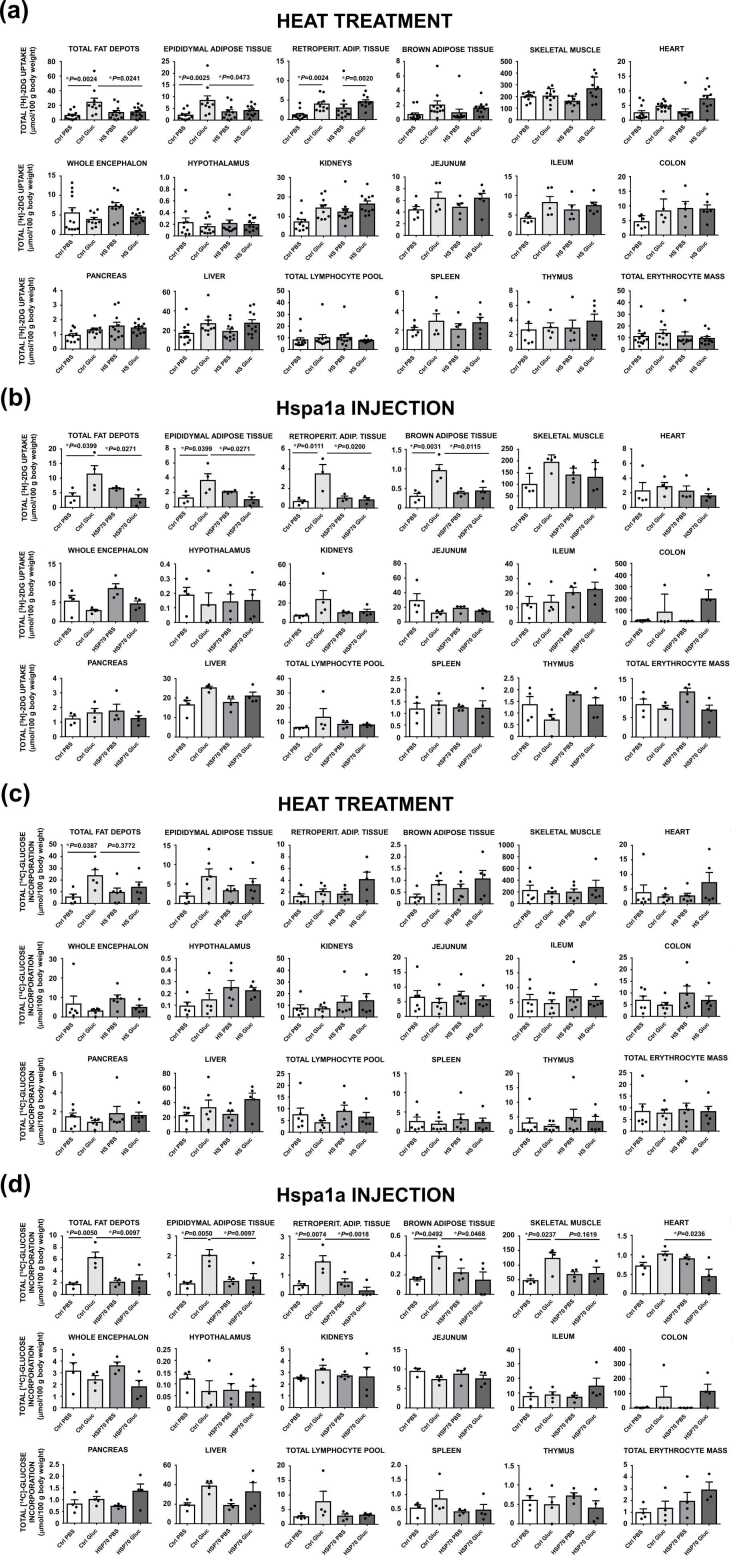
*HS treatment reduces uptake of [^3^H]-2DG and incorporation of [^14^C]-glucose into visceral adipose tissue during ipGTT that is mimicked by hspa1a injection.* The rats were fasted overnight for 12 h from 08:00 pm to 08:00 am, when the HS (or hspa1a injection, sufficient for attaining 139 pM plasma concentration) protocol started. Twelve hours after HS (or hspa1a injection), the animals were submitted to ipGTT in the presence of the radiolabeled tracers vehiculated in 0.8 g/mL cold glucose (1 g/kg body weight). The animals were then killed 40 min after glucose overload (uptake) or after 60 min (incorporations). Simultaneously to blood collection, different tissues were surgically excised and freeze-clamped to be analyzed by liquid scintillation. **(a)** [^3^H]-2DG uptake after HS treatment (*n* = 12) or **(b)** hspa1a injection (*n* = 6). **(c)** [^14^C]-glucose incorporations after HS treatment (*n* = 6) or **(d)** hspa1a injection (*n* = 6). Unnormalised gross (dpm/g tissue) data for [^3^H]-2DG uptake and of [^14^C]-glucose incorporations are presented in Supplementary Table1, while specific uptakes of [^3^H]-2DG or [^14^C]-glucose incorporations (μmol/g tissue) are depicted in Supplementary Figure 6, and the clearances (mL/g or mL/mL of RBC) for [^3^H]-2DG and [^14^C]-glucose are shown in Supplementary Figure 7. Data are presented as the means ± s.d. and were analyzed by 1-way ANOVA followed by Tukey’s multiple comparison testing.

If HS and HSP70 evoke hyperglycemia during ipGTT by impeding insulin action, it is plausible that excess glucose in the circulation could be due to decreased glucose uptake and/or utilization in insulin-dependent tissues. Hence, we performed ipGTT 12 h after HS or HSP70 injection (139 pM) adding either radiolabeled 2-deoxyglucose ([^3^H]-2DG) or [^14^C]-glucose to the cold glucose solution in order to assess tissue glucose uptake and utilization, respectively. The results (Figure 5, Supplementary Table 1, Supplementary Figures 6 and 7) show that HS decreases both uptake and utilization of glucose in fat depots (Figure 5a, c). However, although injection of HSP70 alone does not reproduce the hyperglycemic effect of HS during ipGTT (Figure 3d and e), HSP70 administration does reduce glucose uptake and its utilization in visceral adipose tissue (Figure 5b and d). Despite HSP70 not interfering in glucose management by skeletal muscle, it reduces cardiac glucose utilization (Figure 5d). This prompted us to argue whether HS could influence insulin signaling in skeletal muscle. Nevertheless, *ex vivo* incubations of soleus muscle obtained from rats 12 h post-HS indicate that the response to pharmacological doses of insulin in the presence of 11 mM glucose and dialyzed BSA (instead of real plasma) does not change insulin receptor expression nor its Tyr972-phosphorylated form (Supplementary Figure 8).

We therefore investigated if the soleus muscle *ex vivo* incubated with increasing concentrations of rat/mouse HSP70 could modify 2-DG uptake in the presence or absence of physiological dose of insulin (10 μU/mL = 60 pM, which equals *ca.* 4.5 ng/mL HSP70) in dialyzed autologous rat serum with or without ADP, since ATP promotes the release of the client peptides and ADP maintains them in the active site. Hence, in studies of HSP70 binding to client proteins, ADP is used.^26^

Under control conditions (insulin + serum + ADP), HSP70 reduces 2-DG uptake by *ca.* 40 % (*P* = 0.0147) exclusively at 5.5 mM total glucose (fasting level) but not at 11.0 mM (fed state) or 2.75 mM (hypoglycemia), as shown in Supplementary Figure 9a. This effect persists up to 500 ng/mL HSP70 (not shown). Notably, withdrawing insulin entirely abolishes the HSP70-dependent inhibition of 2-DG uptake in a serum-dependent manner (Supplementary Figure 9b), indicating that the observed changes stem from HSP70–insulin interactions rather than a direct effect of HSP70 on the tissue. At hypoglycemic glucose levels, HSP70 paradoxically enhances insulin-induced 2-DG uptake by *ca.* 50 % at concentrations above 10 ng/mL, even in the absence of serum and ADP (Supplementary Figure 9c). Conversely, at fed-state glucose levels, HSP70 markedly suppresses 2-DG uptake by up to 50 % at ≥5 ng/mL while promoting uptake in the absence of insulin (Supplementary Figure 9d). Whether this unexpected effect arises from HSP70 signaling via Toll-like receptors^27^, ^28^, ^29^ remains under scrutinization.

To further investigate the mechanism underlying HSP70-induced suppression of 2-DG uptake at 5.5 mM glucose, differentiated rat myoblasts were incubated under conditions similar to *ex vivo* studies but without ADP and with dialyzed FBS instead of autologous serum from starved rats. Under these conditions, HSP70 no longer affected 2-DG uptake (Supplementary Figure 10), suggesting that both ADP and an additional serum component (possibly HIP) are required to fully inhibit insulin-induced 2-DG uptake by HSP70.

## Discussion

In the present study, our data support a model in which HSP70 functions as an extracellular chaperone in blood plasma, binding insulin (Figure 1) with high affinity (K_d_ ∼3 pM), thereby modulating its bioavailability to insulin-sensitive tissues (Figure 5). This finding challenges the prevailing view that HSP70 chaperone function is restricted to the intracellular milieu. Accordingly, under stress conditions (e.g., HS, prolonged fasting, exercise), elevated plasma HSP70 may enhance glucose availability, with effects lasting over 12 h.

Although the affinity estimated for the HSP70-insulin interaction lies within the picomolar range—exceeding that reported for most classical hormone-receptor systems—this observation should be interpreted with caution, as the current methodology does not fully resolve binding stoichiometry or kinetics and may reflect a composite, potentially cofactor-dependent interaction in the plasma milieu. Please see Limitations and Open Questions section.

HS increases peak glycemia in fasting animals subjected to ipGTT by ∼2.7 mM (Figure 2**a**), a clinically significant ∼50 mg/dL rise. This hyperglycemic effect, evident 12 h post-HS during the glucose-induced insulin peak (Supplementary Figure 3a), is HIP-dependent (Figure 3i, j) and is abolished by anti-HSP70 monoclonal antibody (mAb) (Figure 3**a**), supporting a role for extracellular HSP70 in this response. In contrast, the effect is not altered by quercetin (Figure 3c, Supplementary Figure 5), suggesting that it arises from stress-induced exosomal release of pre-assembled HSP70 rather than *de novo* synthesis. Exosomal HSP70 trafficking has been reported in leukocytes,^4^, ^7^ endothelial^30^ and tumor cells,^31^, ^32^ among others.^8^ On the other hand, extracellular vesicle (EV)-associated and membrane-bound HSP70 are well documented in tumor biology, immune modulation, and intercellular signaling, including roles in tumor cell migration and invasiveness.^32^, ^33^, ^34^ Notably, in cancer patients, circulating exosomal HSP70 levels—but not soluble HSP70—have been shown to reflect tumor HSP70 content,^35^ indicating that vesicular and soluble HSP70 pools may have distinct biological roles, including differences in cellular uptake and intercellular signaling. This distinction further supports the need to consider extracellular HSP70 heterogeneity in different physiological and pathological contexts.

While we cannot exclude a contribution of EV-associated HSP70—particularly given that HSP70 can be exported both as a soluble protein and in association with EVs^32^—our findings are more consistent with a readily accessible soluble fraction mediating this interaction. This interpretation is supported by the rapid kinetics of the observed effects, which are more compatible with a pre-existing circulating pool than with mechanisms requiring vesicle trafficking, membrane docking, and release. Consistent with this view, Supplementary Figure 3 shows a rapid increase in circulating HSP72 (HSPA1A/HSPA1B) and HSP73 (HSPA8), accompanied by a transient impairment in glucose tolerance immediately following the HS session (Figure 2). Nevertheless, the relative contribution of vesicular versus soluble HSP70 to insulin binding remains to be determined.

Intriguingly, plasma HIP levels decrease in fasting animals subjected to glucose overload–induced hyperinsulinemia combined with HS (Figure 3g). This observation raises the possibility that HIP may associate with excess circulating insulin and/or HSP70–insulin complexes in plasma. Whether HIP contributes to insulin neutralization in the circulation and whether it is co-secreted with HSP70 into the extracellular milieu remains to be determined.

We further infer that HSP73 (hspa8), the constitutive housekeeping HSP70 paralogue, is likely to be the major extracellular HSP70 species contributing to hyperglycemia. Its plasma levels (2156 pM) 12 h post-HS exceed those of HSP72 (hspa1a/hspa1b) by >30-fold (Supplementary Figure 3a), consistent with our previous findings in HS and exercise.^4^, ^36^ Given plasma insulin levels of 296-789 pM (∼49-132 μU/mL) at this timepoint, HSP73 could, in molar terms, sequester up to 7.3 times the total circulating insulin.

Notably, HS induces a surge in insulin during ipGTT (Supplementary Figure 3a), with a modest increase in the insulinogenic index, thereby excluding hypoinsulinemia as a cause of hyperglycemia. Insulin receptor expression and downstream signaling remain intact 12 h post-HS (Supplementary Figure 8), indicating that the observed effects are more consistent with reduced insulin bioavailability rather than with impaired receptor signaling. Despite impaired glucose tolerance at 12 h (Figure 2**a**), insulin sensitivity improves 24 h post-HS during ipITT (Figure 2**b**), aligning with previous reports that both acute^37^ and chronic heat exposure^13^, ^38^ improve glucose tolerance and reduce insulin resistance via intracellular HSP70 and reduced JNK activity in skeletal muscle.^13^, ^38^, ^39^

At the molecular level, post-translational modifications (PTMs) of HSP70, including phosphorylation, have emerged as important regulators of chaperone function and client interactions, contributing to the so-called “chaperone code."^40^ Although we did not directly assess PTMs in the present study, the rapid kinetics of the observed effects, together with the lack of impact of quercetin-mediated inhibition of HSP70 expression, suggest that the extracellular actions described here primarily involve pre-existing pools of HSP70 rather than newly synthesized or extensively modified protein. Nevertheless, it remains possible that specific PTMs may modulate the affinity or stability of HSP70–insulin complexes, and this should be addressed in future studies.

Heat stress induces a coordinated response involving multiple HS proteins organized within an integrated chaperone network (“chaperome”), in which the HSP70 and HSP90 families may play central and interconnected roles in cellular proteostasis and stress adaptation.^41^ Recent advances in the development of isoform-selective HSP90 inhibitors further highlight the functional and therapeutic relevance of this system.^42^

Although the present study focused on HSP70, it is possible that other chaperones, such as HSP60 and HSP90, are also present in the extracellular milieu following stress. However, the complete abrogation of the hyperglycemic phenotype by anti-HSP70 monoclonal antibody treatment supports a primary functional role for HSP70 in the mechanism described here.

Taken together, these findings support a model in which extracellular HSP70 modulates insulin bioavailability under acute stress conditions, thereby contributing to transient alterations in glycemic control. While this framework is grounded in the present experimental data, its broader implications for metabolic disease require careful consideration.

Reflecting its role in maintaining glycemic homeostasis, chronic degenerative inflammatory diseases in humans and laboratory animals consistently exhibit suppressed expression of intracellular HSP70 and its associated HSR in adipose tissue, skeletal muscle, liver, pancreas, vascular beds, and the central nervous system. Suppressed HSR, a potent intracellular anti-inflammatory pathway, undermines the resolution of inflammation, fostering chronic low-grade inflammatory conditions,^14^, ^15^ as observed in the skeletal muscle of type 2 diabetic patients,^13^, ^43^, ^44^ in the adipose tissue and liver of patients with nonalcoholic fatty liver disease-NAFLD (or metabolic dysfunction-associated steatotic liver disease-MASLD),^45^ and in the vascular beds of individuals with atherosclerosis.^21^, ^46^

Similar impairments occur in menopause-related metabolic dysfunctions,^47^, ^48^ as well as in aging primates and humans, including cases with neurodegenerative diseases.^49^, ^50^, ^51^, ^52^, ^53^ Clinically, HSR failure combined with insulin resistance can be detected by HSP70 expression in whole-blood heat-stressed leukocytes, offering a potential early diagnostic marker of obesity-driven inflammation and glycemic dysregulation before any clinical manifestation.^54^

Interventions that enhance intracellular HSP70 (e.g., HS, exercise, pharmacological approaches) improve glucose tolerance and reduce insulin resistance, with some reversing atherosclerosis.^13^, ^21^, ^37^, ^38^, ^55^, ^56^, ^57^ HS benefits were first reported by Hooper,^58^ who observed reduced fasting glycaemia, HbA_1c_, and body weight in diabetic patients submitted to hot tubbing.^58^ Chronic HS ameliorates immunoinflammatory status and the proliferative senescence of adipose tissue,^59^ while preventing insulin resistance-induced vascular complications in rodents.^60^ Meta-analyses have confirmed the benefits of HS therapy in humans.^61^, ^62^

In this context, it is noteworthy that even brief HS (31 °C) improves insulin sensitivity in healthy humans while transiently impairing glucose tolerance, leading to increases in both glycemia and insulinemia.^63^ This response has been attributed to augmented hepatic glucose production driven by sympathetic activation. In fact, HS induces a hyperadrenergic state in humans, elevating plasma adrenaline,^20^, ^64^ while exercise similarly promotes α-adrenergic stimulation of the hepatosplanchnic region, triggering HSP70 release into the bloodstream.^2^ Consistent with this framework, a similar (∼2 mM) HS-dependent hyperglycemic effects have been observed in rodents,^13^ non-human primates,^65^ and healthy volunteers.^64^

Our previous observations^66^ that intravenous injection of non-activated allogeneic lymphocytes induces a hyperinsulinemic hyperglycemic response without any depletion of hepatic glycogen content further support the notion that rapid increases in circulating HSP70 may contribute to transient dysregulation of glucose homeostasis. Because lymphocytes are a major source of extracellular HSP70,^3^ these findings are consistent with the present model. Although adrenergic-dependent glycogenolysis may contribute, our current data are more consistent with an HSP70/HIP-dependent mechanism underlying the glucose intolerance observed after HS, as evidenced by its abrogation following anti-HSP70 mAb treatment (Figure 3). The potential contribution of hepatic glycogenolysis and gluconeogenesis to the HS-induced hyperglycemic response is currently under investigation in our laboratory.

Although the present findings support a primary role for extracellular HSP70 in modulating insulin bioavailability, we cannot exclude contributions from downstream intracellular mechanisms. In particular, changes in glucose transporter dynamics (e.g., GLUT4 translocation in skeletal muscle and adipose tissue or GLUT2 expression in the liver) and insulin signaling pathways may contribute to the metabolic phenotype observed following HS. However, the rapid normalization of glycemic responses following anti-HSP70 monoclonal antibody treatment, without direct manipulation of intracellular pathways, is more consistent with an upstream mechanism involving extracellular insulin sequestration.

While intracellular HSP70 exerts well-established cytoprotective and anti-inflammatory functions, its extracellular counterpart acts as a pro-inflammatory signal, engaging TLR2 and TLR4 in a CD14-dependent manner and activating MyD88/IRAK/NF-κB pathways.^14^, ^15^, ^27^, ^28^, ^67^ Elevated plasma HSP70 levels correlate with insulin resistance in vivo^28^, ^68^ and are associated with β-cell dysfunction in vitro,^28^ as well as with metabolic and inflammatory conditions such as NASH/MASH and critical illness,^28^, ^69^, ^70^, ^71^, ^72^, ^73^, ^74^, ^75^, ^76^ which are accompanied by increased visceral adipose tissue and compensatory insulin secretion.^77^

It is important to emphasize that the present findings are derived from an acute HS model in rats and therefore do not directly address the complex pathophysiology of chronic metabolic diseases in humans. Nevertheless, collectively, these observations suggest that extracellular HSP70 may integrate inflammatory and metabolic signals, linking stress responses to insulin availability and glycemic control. In this framework, the interaction between extracellular HSP70 and insulin may represent a previously unrecognized mechanism contributing to hyperinsulinemia and metabolic dysregulation in chronic disease states. These observations raise the possibility that targeting extracellular HSP70 with humanized anti-HSP70 mAb^78^, ^79^ may represent a strategy to modulate metabolic inflammation and insulin availability under conditions of chronic metabolic stress. The binding of HSP70 to insulin may also explain the hyperglycemia observed in septic patients with poor prognoses, who exhibit markedly elevated plasma HSP72 levels,^80^, ^81^ thus highlighting a potential therapeutic role for anti‑HSP70 mAb. However, such therapeutic implications remain speculative and will require direct experimental validation. Of note, experimental studies in animal models have shown that neutralization of extracellular HSP70 improves insulin resistance and metabolic dysfunction under high-fat diet conditions,^68^, ^77^ although the underlying mechanisms remain to be fully elucidated.

In considering the potential translational application of this approach, several safety and pharmacological aspects warrant careful attention. The proposed strategy is based on the selective targeting of extracellular HSP70, which is markedly elevated under pathological conditions. Indeed, circulating HSP70 levels in chronic inflammatory states can reach the nanogram per milliliter range (corresponding to low nanomolar concentrations), substantially exceeding basal physiological levels. Under such conditions, equimolar or near-equimolar dosing of monoclonal antibodies would be expected to preferentially neutralize the excess extracellular HSP70 pool. Moreover, given the abundance of circulating HSP70, a substantial fraction of administered antibodies would likely be engaged in extracellular binding, potentially limiting their intracellular distribution. Nevertheless, intracellular effects cannot be excluded, particularly in light of evidence that antibodies targeting membrane-associated HSP70 may undergo internalization, and this possibility should be carefully evaluated in future studies.

In addition, experimental evidence supports the feasibility of targeting extracellular HSP70 in vivo. Chronic infusion of anti-HSP70 monoclonal antibodies in high-fat diet models reverses insulin resistance, hyperglycemia, NAFLD, and obesity^68^, ^77^ and anti-HSP70 mAb treatment has been shown to attenuate inflammatory signaling and improve cardiac function in models of heart failure.^79^ In this context, the use of humanized anti-HSP70 monoclonal antibodies, as described by Zettlitz et al.,^78^ may reduce immunogenicity and improve translational applicability. However, the underlying mechanisms, pharmacokinetic behavior, tissue distribution, and long-term safety of such interventions remain to be established. Moreover, the potential immunogenicity of monoclonal antibody-based interventions should be considered. The development of humanized anti-HSP70 monoclonal antibodies^78^ may mitigate immune responses and improve translational applicability. Nevertheless, the immunological consequences, as well as systemic and tissue-specific effects of such approaches, remain to be fully characterized.

The apparent paradox between the transient impairment in glucose tolerance observed shortly after HS and the subsequent improvement in insulin sensitivity at later time points likely reflects the coexistence of temporally and mechanistically distinct processes. Acutely, HS induces a rapid increase in circulating HSP70, which interacts with insulin in the extracellular compartment, reducing its immediate bioavailability and thereby transiently reducing effective glucose disposal during ipGTT. This effect is consistent with a counterregulatory mechanism operating under acute stress conditions, in which limiting insulin action may prevent excessive glucose clearance and potential hypoglycemia during hyperadrenergic states. In contrast, at later time points (e.g., 24 h after HS), activation of the intracellular heat shock response (HSR) leads to increased expression of cytoprotective HSP70, attenuation of inflammatory signaling pathways, and improved insulin sensitivity. Thus, extracellular and intracellular HSP70 appear to exert functionally distinct and temporally coordinated roles, with the former modulating acute insulin availability and the latter contributing to longer-term metabolic adaptation.

**Fig. 6:**
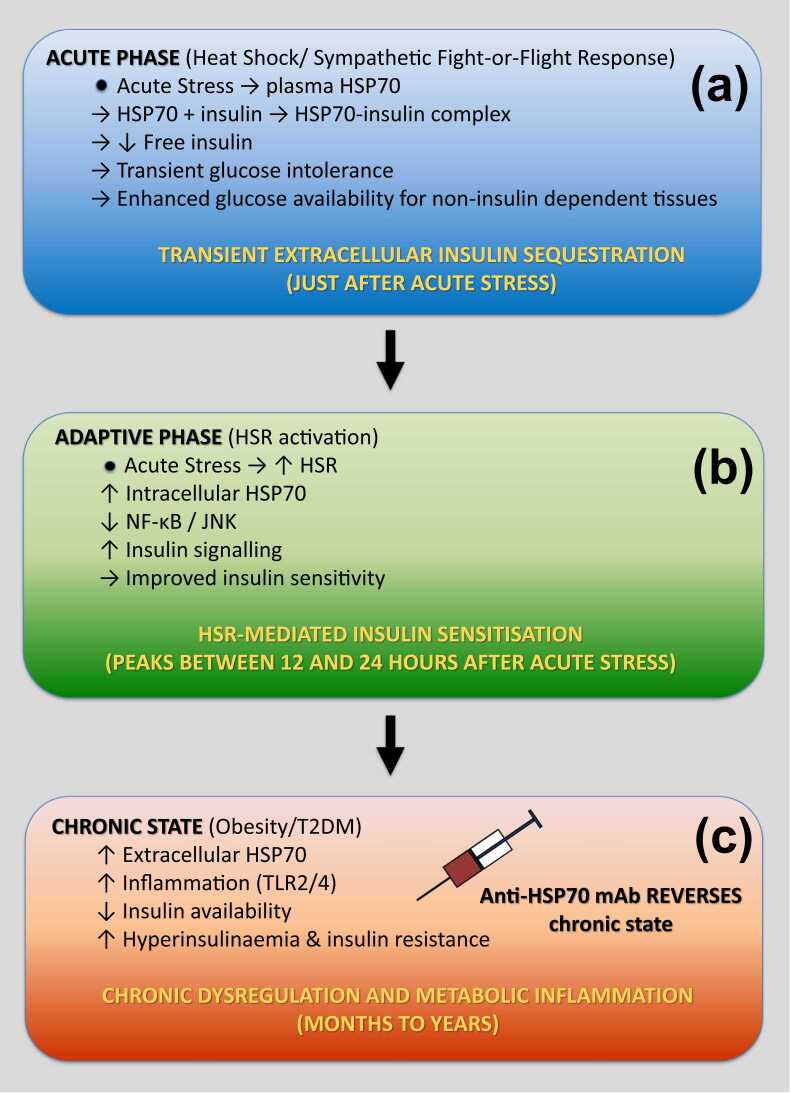
*Temporal model of HSP70–insulin interaction: extracellular versus intracellular effects.***(a)** Transient extracellular insulin sequestration; **(b)** delayed intracellular phase: heat shock response (HSR)-mediated insulin sensitization; **(c)** chronic dysregulation and metabolic inflammation.

This dual-phase model may also provide a conceptual framework for understanding the transition from physiological adaptation to pathological dysregulation (Figure 6). Under acute stress, transient elevations in extracellular HSP70 may serve a protective role by buffering insulin action. However, under chronic conditions such as obesity and type 2 diabetes, sustained elevations of extracellular HSP70—together with persistent inflammatory signaling—may contribute to reduced insulin bioavailability and compensatory hyperinsulinemia, thereby exacerbating insulin resistance. In this context, extracellular HSP70 may act as a previously unrecognized integrator of stress and metabolic signaling across different temporal scales.

Beyond these considerations, the potential impact of extracellular HSP70 neutralization on hyperinsulinemia merits attention. Chronic hyperinsulinemia is itself a driver of insulin resistance,^82^, ^83^, ^84^, ^85^, ^86^ through feedback mechanisms that attenuate insulin signaling, including receptor internalization and activation of pro-inflammatory pathways such as JNK.^84^, ^87^, ^88^, ^89^, ^90^ In hyperinsulinemic states, augmented receptor internalization reduces receptor density, thus compelling pancreatic β-cells to increment insulin production, ultimately predisposing them to failure—a process evident in advanced type-2 diabetes.^28^, ^91^, ^92^, ^93^ In this context, elevated circulating HSP70 may further aggravate metabolic dysfunction by sustaining both inflammatory signaling and reduced insulin bioavailability, which could stimulate hypersecretion of insulin.

Neutralization of extracellular HSP70 could therefore exert a dual beneficial effect: attenuating inflammation-driven insulin resistance and restoring effective insulin action by increasing the availability of free hormone to target tissues, thereby reducing the need for compensatory hyperinsulinemia. Consistent with this rationale, experimental studies indicate that reducing hyperinsulinemia confers metabolic protection and improves insulin sensitivity.^85^, ^86^ While these considerations remain speculative, they provide a mechanistic framework for future studies exploring extracellular HSP70 as a therapeutic target in chronic metabolic disease.

## Conclusion

Overall, we show that HSP70 sequesters insulin in an HIP-dependent manner. While classic extracellular chaperones protect proteins from the hostile plasma environment,^94^ our findings reveal a novel role for HSP70 as a plasma chaperone and identify HIP as an extracellular cofactor for the first time. Although further mechanistic studies are warranted to clarify the roles of HSP70, HIP, and other potential co-chaperones, our results provide clear and reproducible evidence: co-administration of HSP70 and HIP mimics the effect of HS on glycemic curves, and, importantly, administration of an anti-HSP70 monoclonal antibody completely abolishes HS-induced glucose intolerance. In addition, we demonstrate that insulin co-immunoprecipitates with HSP70 and vice versa, supporting a direct and functionally relevant interaction between insulin and HSP70, despite the existence or not of any specific unknown mechanism. Furthermore, to our knowledge, this is the first report of HIP in plasma, where it may cooperate with extracellular HSP70. In line with its known intracellular function, HIP could contribute to stabilizing the HSP70-insulin complex by prolonging substrate binding,^23^ a possibility that will be further explored in ongoing mechanistic studies in our laboratory.

Within the cell, HIP facilitates HSP70 loading by binding near the nucleotide-binding region, stabilizing its ADP-bound state, and inhibiting its ATPase cycle, thereby increasing substrate affinity. Initially described as a ‘presequence binding factor’ of unknown identity,^95^ HIP is now recognized as a crucial co-chaperone in the HSP70 machinery, protecting proteins across different organelles.^96^ Although no extracellular chaperone function of HIP has been reported to date, our detection of HSP70 and HIP—without other co-chaperones—suggests that they may also act together outside the cell as a continuum of their intracellular roles.

The selective detection of HIP, in contrast to other co-chaperones screened, may reflect functional specialization in the extracellular milieu. While J-domain proteins (DNAJs) primarily facilitate ATP-dependent substrate processing within the canonical intracellular chaperone cycle,^97^ HIP stabilizes the ADP-bound state of HSP70, thereby prolonging client binding. In the context of extracellular interactions involving already folded proteins such as insulin, a stabilizing co-chaperone may be more functionally appropriate than one promoting ATPase cycling. Alternatively, selective vesicular export or limited detectability of other co-chaperones may also contribute to this observation.

A limitation of our study, however, is the challenge of experimentally reproducing the full complexity of plasma composition and sources, which may modulate the HSP70-HIP-insulin interaction. Thus, extracellular chaperone activity should be regarded as being at a similar stage as extracellular HSP signaling – an emerging field now set for exploration in cell-to-cell and organ-to-organ communication.^98^

Finally, it is important to highlight that the protocols used were not intended to establish causality in the strict, interventional sense. Our objective was mechanistic: to test whether extracellular HSP70, in the presence or absence of some co-chaperone (HIP, in the present case), binds insulin with high affinity and thereby modulates glycaemia. Within that scope, our data provide convergent evidence consistent with a causal mechanism:

1. *Temporality and physiology:* HS precedes the rise in plasma HSP70, which in turn precedes an ∼3 mM increase in glycemia at 30 min during ipGTT in fasted rats.
2. *Specific mechanism:* We show an apparent *high-affinity binding* of HSP70 to insulin (K_d_ ≈ 3 pM) and *HIP-dependence* of the HS-induced glucose intolerance (peaking at 12 h), while *insulin receptor signaling remains unaffected*, indicating sequestration rather than receptor antagonism.
3. *Biological coherence:* Glucose ingestion is known to *block exercise-induced HSP70 secretion*, aligning with our model in which extracellular HSP70 availability governs the extent of insulin sequestration and transient glucose intolerance.

Based upon the present results, we propose that, under acute stress, HSP70 released into the circulation via an adrenoreceptor-dependent pathway^2^, ^9^, ^20^, ^64^ may serve as a counterregulatory mechanism by binding insulin and thereby limiting its bioavailability, protecting against potentially dangerous hypoglycemia. This is particularly relevant given that insulin has a short half-life (∼5 min),^18^, ^19^ yet measurable circulating insulin—and, more importantly, its biological effects—may persist for extended periods before complete clearance. Under such conditions, a rapid counterregulatory mechanism becomes essential to prevent potentially dangerous hypoglycemia during acute stress responses, whereas circulating HSP70 persists for longer periods (∼60 min).^77^

Moreover, once insulin binds to its receptor, the downstream metabolic effects—mediated through intracellular signaling cascades—may persist for extended periods, even after the hormone has been cleared from the circulation. In this context, modulation of insulin bioavailability at the extracellular level may represent an anticipatory and more efficient regulatory strategy than attempting to counteract already established intracellular signaling. Given that circulating HSP70 is present at molar concentrations that exceed those of insulin, its capacity to sequester the hormone may constitute an evolutionarily advantageous mechanism to rapidly fine-tune glycemic control under conditions of acute stress.

This mechanism may allow for safer and more sustained glycemic regulation during hyperadrenergic states, as evidenced by the ∼3 mM increase in plasma glucose observed during ipGTT 12 h after HS. We are currently investigating whether HSP70 also binds other peptide hormones, further expanding its role in endocrine regulation.

## Limitations and open questions

The present study was designed to test a mechanistic hypothesis, namely whether extracellular HSP70, in the presence or absence of co-chaperones such as HIP, binds insulin with high affinity and modulates its bioavailability *in vivo*. While our findings provide consistent evidence supporting this model, several limitations and open questions should be acknowledged.

### Mechanistic and molecular limitations

#### Co-chaperone specificity and HIP

The selective detection of HIP should be interpreted with caution, as it may reflect functional specialization, selective extracellular export, or methodological limitations in detecting other co-chaperones under the present experimental conditions. Accordingly, the selective detection of HIP, in contrast to other co-chaperones such as DNAJA1, HSPB6, BAG1, and HspBP1, is an intriguing observation that warrants consideration. At present, we can only offer plausible interpretations. One possibility relates to the functional specialization of co-chaperones. While J-domain proteins (DNAJ family) are primarily involved in stimulating the ATPase activity of HSP70 and promoting substrate loading within the canonical intracellular chaperone cycle,^97^ HIP operates differently. By binding near the nucleotide-binding domain of HSP70, HIP stabilizes the ADP-bound state and inhibits ATP hydrolysis, thereby increasing substrate retention and affinity.^96^, ^98^ In this context, if the extracellular function of HSP70 involves stabilizing interactions with already folded client proteins—such as circulating insulin—rather than facilitating protein folding or degradation, a co-chaperone that prolongs substrate binding would be more functionally appropriate than one that accelerates ATPase cycling. This interpretation is consistent with our experimental and *in silico* data, which support a stabilizing role of HIP in the HSP70–insulin interaction.

A second, non-exclusive possibility is that extracellular export be selective. If circulating HSP70 is released, at least in part, via EVs or stress-associated vesicular pathways, the associated co-chaperone cargo may not reflect the full intracellular repertoire. Under such conditions, HIP may be preferentially co-exported, whereas DNAJA1 and other co-chaperones may be absent, rapidly cleared, or remain below the detection threshold in plasma.

Finally, we cannot exclude methodological limitations. The absence of detectable DNAJA1, BAG1, HSPB6, or HspBP1 does not necessarily indicate their complete absence in the extracellular compartment, but rather that they were not detected under the present experimental conditions.

### Experimental and methodological limitations

#### Acute model and generalizability

The present experimental framework relies primarily on acute HS as a physiological stimulus to increase circulating HSP70. Although HS, exercise, and fasting share common stress-related pathways, the extent to which the present findings can be generalized to other forms of metabolic stress remains to be established. Ongoing studies in our laboratory are evaluating the effects of exercise, fasting alone, adrenergic stimulation, and pharmacological modulators of HSP70 expression (e.g., glutamine, BGP-15) on both acute and chronic glycemic control. In addition, it will be important to determine whether similar HSP70–insulin interactions occur under chronic metabolic conditions such as obesity, T2DM, and nonalcoholic steatohepatitis (NASH/MASH).

#### Structural modeling and binding mechanism

While molecular docking and MD simulations provided a structural framework supporting the interaction between HSP70 and insulin, these analyses should be interpreted with caution. The models were generated using established force fields and simulation protocols and demonstrated stable interaction patterns over time; however, they were not designed to yield a fully validated high-resolution structural model.

An important conceptual limitation arises from the fact that the SBD of HSP70 is classically involved in the recognition of unfolded or partially folded polypeptides, whereas insulin is a compact, folded peptide hormone. Thus, the interaction proposed here may involve non-canonical binding modes or context-dependent conformational flexibility, potentially influenced by plasma components or co-chaperones such as HIP. Furthermore, the possibility that insulin binding occurs within higher-order or multimeric HSP70 complexes cannot be excluded, which may further complicate the interpretation of docking-derived interfaces.

Future studies will be required to experimentally validate the predicted interaction surfaces. In particular, site-directed mutagenesis of residues within the HSP70 SBD—especially those clustered in the Leu403–Ala412, Gly437–Glu444, and Thr450–Ser462 regions identified by docking and MD—should help determine whether these segments are required for insulin recognition. Reciprocal mutational analyses targeting the insulin regions Phe1–Leu17 and Cys39–Leu45 may likewise clarify the relative contribution of the hormone surface to complex stability. These experiments should be complemented by binding assays using purified proteins and orthogonal biophysical approaches, such as surface plasmon resonance, microscale thermophoresis, or calorimetric methods, in order to define binding kinetics, affinity, and stoichiometry under controlled conditions. Such studies will be critical to establish whether the interaction observed here reflects a direct and specific binding event or a more complex, context-dependent association within the extracellular environment.

In this setting, we believe that the computational analysis provides a coherent structural framework that complements the experimental findings while clearly recognizing its limitations and the need for future validation.

#### Quantitative behavior and plasma-dependent interaction

Although we demonstrate high-affinity binding between HSP70 and insulin in plasma, the precise molecular determinants of this interaction remain to be elucidated. It is not yet clear whether HSP70 directly competes with the insulin receptor for binding, whether additional plasma components modulate this interaction, or whether co-chaperones other than HIP contribute to complex stability. The role of extracellular HIP, identified here for the first time in blood plasma, warrants further investigation, including its origin, secretion dynamics, and functional specificity in the extracellular milieu.

An additional unresolved issue concerns the quantitative behavior of the HSP70–insulin equilibrium under physiological conditions. At the time point of maximal glucose intolerance during ipGTT performed 12 h after HS, circulating insulin reached 789.4 pM, whereas total measurable circulating HSP70 species amounted to approximately 390.7 pM, considering both HSP72 (132.4 pM) and HSP73 (258.3 pM). Thus, even under a conservative 1:1 binding assumption, the measured HSP70 concentrations would be sufficient to sequester a substantial fraction of the circulating insulin pool. Given the apparent picomolar affinity estimated in the present study, such an interaction is quantitatively plausible and could significantly reduce free insulin bioavailability.

However, HSP70 abundance alone does not appear to determine insulin sequestration. In the absence of glucose overload, circulating HSP73 reached approximately 2156 pM 12 h after HS—approximately threefold higher than basal insulin concentrations—yet insulin remained detectable and biologically active. This indicates that the presence of HSP70 in molar excess is not, by itself, sufficient to neutralize circulating insulin.

Consistent with this, recombinant HSP70 alone, even at nanomolar concentrations, failed to reproduce the hyperglycemic phenotype induced by HS, suggesting that additional plasma components are required. In this context, the detection of HIP in plasma, together with its reduction under hyperinsulinemic conditions, raises the possibility that the effective extracellular equilibrium involves co-factor dependence, multivalent interactions, and/or higher-order complexes.

Future studies combining direct biophysical measurements with quantitative modeling will therefore be necessary to define more precisely the stoichiometry and dynamics of insulin sequestration in plasma. Additionally, the role of HIP in the extracellular environment also remains incompletely characterized. Although HIP was consistently detected in plasma and functionally contributed to the metabolic effects observed *in vivo*, its physiological concentration, cellular origin, and mechanisms of release remain unknown. The identification of HIP as a circulating protein emerged at a late stage of the present study and, because of this, no quantitative assays are currently available to determine its plasma levels.

Furthermore, it remains to be established whether HIP is present as a freely soluble protein or associated with EVs. Given previous evidence that HSP70 can be released via exosome-associated pathways under HS conditions,^4^ it is conceivable that HIP may follow similar routes; however, this hypothesis requires direct experimental confirmation. Future studies should therefore address the quantification, cellular source, and extracellular trafficking of HIP, as well as its precise role in modulating HSP70-dependent insulin binding.

#### Soluble versus vesicular HSP70

Another important open question concerns the molecular form in which extracellular HSP70 mediates its interaction with insulin. HSP70 can be present both as a freely soluble protein and associated with EVs, and these distinct pools may exert different biological functions, particularly in pathological contexts such as cancer.^32^, ^33^, ^34^, ^99^ While we cannot exclude a contribution of EV-associated HSP70, our findings are more consistent with a readily accessible, likely soluble fraction mediating this interaction. This interpretation is supported by the rapid kinetics of the observed effects, which are more compatible with a pre-existing circulating pool than with mechanisms requiring vesicle trafficking, membrane docking, and release.

Consistent with this view, we observed a rapid increase in circulating HSP70 forms immediately following HS, accompanied by transient glucose intolerance. Notably, the constitutive paralogue HSP73 (hspa8) predominated in plasma, with concentrations substantially higher than those of the stress-inducible HSP72 isoforms, suggesting that “constitutive” housekeeping HSP70 may represent the major circulating pool involved in this interaction. Similar patterns have been reported in response to exercise,^36^ supporting the generalizability of this observation across physiological stressors. Nevertheless, distinguishing between soluble and vesicular HSP70, as well as determining their relative contributions to insulin binding, will require dedicated experimental approaches, including EV isolation, fractionation strategies, and functional assays.

Importantly, our quantitative data indicate that hspa8 represents the predominant circulating HSP70 isoform under our experimental conditions, both immediately and at later time points following HS. As shown in Supplementary Fig 3, circulating hspa8 levels were markedly higher than those of hspa1a/hspa1b (approximately 32-fold), indicating that the majority of extracellular HSP70 detected in plasma corresponds to the constitutive isoform. Therefore, while the antibody used for immunoprecipitation does not distinguish between individual HSP70 paraloges, the experimental evidence indicates that hspa8 is the major species contributing to the observed interaction. Importantly, the central finding of this study—namely that extracellular HSP70 associates with insulin in plasma—remains valid irrespective of the specific paralogue involved. In anyway, future studies employing isoform-specific antibodies (e.g., selective for HSP72 vs HSP73) will be important to determine whether different HSP70 family members differentially contribute to insulin binding and metabolic regulation.

Our data support a model in which extracellular HSP70 reduces insulin bioavailability without impairing receptor signaling. However, quantitative assessment of insulin distribution between bound and free fractions *in vivo*, as well as kinetic analyses of complex formation and dissociation under physiological conditions, will be required to fully characterize this mechanism.

#### Methodological limitations of binding analysis

A key limitation of the present study lies in the methodological approach used to characterize the interaction between HSP70 and insulin. Binding parameters were derived from immunoprecipitation-based assays combined with Scatchard and Eadie–Hofstee analyses performed in dialyzed plasma, which, although preserving a physiologically relevant environment, may be influenced by non-specific interactions, antibody interference, or multivalent binding and therefore do not fully resolve binding kinetics or stoichiometry.

In this context, the absence of orthogonal, label-free biophysical validation—such as surface plasmon resonance (SPR), isothermal titration calorimetry (ITC), or microscale thermophoresis (MST)—represents an important limitation. Notably, the affinity estimated in the present study falls within the picomolar range, exceeding that reported for most classical hormone–receptor interactions involved in glycemic regulation, such as insulin (∼0.1-5 nM),^100^, ^101^ glucagon (∼1-10 nM),^102^ and GIP (∼1-10 nM),^103^ while approaching the higher-affinity spectrum observed for incretin and growth factor systems, including GLP-1 and IGF-1.^104^, ^105^, ^106^ Although such high-affinity interactions are uncommon, they are not without precedent.

In the present study, our primary objective was to test a mechanistic hypothesis in a physiological context, namely whether extracellular HSP70 modulates insulin bioavailability under stress conditions. To this end, we combined functional *in vivo* experiments, plasma-based binding assays, co-immunoprecipitation, and MD simulations. Together, these approaches provide convergent—albeit indirect—evidence supporting the existence of an interaction in the extracellular milieu. We acknowledge, however, that we did not directly measure binding kinetics or stoichiometry using purified proteins in solution (e.g., SPR, MST, or ITC). This represents an important limitation that should be considered in further studies.

It is important to note that our data suggest that the interaction may be plasma-dependent and potentially mediated by co-factors such as HIP, as well as by multivalent or higher-order complex formation. In this context, measurements performed in simplified reconstituted systems may not fully recapitulate the interaction observed in plasma, and careful experimental design will be required to preserve relevant molecular states.

Future studies employing orthogonal biophysical approaches in solution will therefore be essential to independently validate the interaction, define its kinetic and thermodynamic parameters, and determine the contribution of HIP and other plasma components to complex formation. This is particularly relevant given that the apparent high affinity observed in the present study may reflect avidity within a dynamic, higher-order complex rather than a simple bimolecular interaction.

We believe that, despite the absence of direct biophysical measurements, the combination of functional evidence (including the rapid modulation of glycemic responses and the effects of HSP70 neutralization), co-immunoprecipitation data, and structural modeling provides a consistent framework supporting the proposed interaction, while clearly defining the need for further validation.

Importantly, our findings suggest that the HSP70–insulin interaction may depend on plasma components, potentially including co-factors such as HIP, and may therefore not be fully recapitulated in simplified *in vitro* systems. In addition, the chaperone nature of HSP70, together with its well-established capacity for oligomerization and multivalent client interactions, raises the possibility that the observed affinity reflects a composite binding process rather than a simple bimolecular interaction.

This interpretation is further supported by MD simulations, which identified two distinct insulin-binding sites within the HSP70–HIP complex, including one located in the substrate-binding domain (SBDα) region implicated in oligomerization interfaces.^25^ Consistent with this notion, co-immunoprecipitation experiments in dialyzed plasma revealed higher molecular weight HSP70-immunoreactive species (∼216 kDa and ∼236 kDa), compatible with multimeric assemblies, potentially involving trimeric HSP70 structures associated with insulin.

Although these observations do not allow definitive assignment of stoichiometry, they support the existence of higher-order complexes in which insulin may engage HSP70 in a cooperative or multivalent manner, potentially involving co-chaperones such as HIP. In particular, simulations incorporating HSP70, HIP, and insulin of rat origin revealed the emergence of multiple binding sites on HSP70 and increased binding energies when insulin and HIP were present simultaneously, suggesting cooperative stabilization of the complex. In this context, the apparent picomolar affinity may reflect the avidity of a dynamic, plasma-dependent assembly rather than the intrinsic affinity of a single binding interface.

Future studies employing cross-linking approaches, native mass spectrometry, or single-particle techniques will be required to resolve the stoichiometry and structural organization of these complexes more precisely.

In addition, we acknowledge that we did not directly assess whether HIP co-immunoprecipitates with insulin or with HSP70–insulin complexes. This represents an important limitation. The identification of HIP as a potential co-factor emerged at a later stage of the study, when we observed that recombinant HSP70 alone did not fully reproduce the metabolic effects induced by HS. This prompted a targeted screening of candidate co-chaperones and interacting proteins in plasma, in which HIP was the only molecule consistently detected. However, at that stage, the *in vivo* experimental series had already been completed, and no additional plasma samples were available to perform co-immunoprecipitation assays.

Although we did not directly demonstrate co-immunoprecipitation, our MD simulations support a structural interaction between insulin and the HSP70–HIP complex, with specific residues in HIP contributing to insulin binding. These findings raise the possibility that HIP may participate in stabilizing or modulating the HSP70–insulin interaction in the extracellular milieu.

With regard to the origin of extracellular HIP, this remains unknown. To our knowledge, there are currently no studies addressing whether HIP is actively released under HS conditions or identifying its cellular source(s). Given that HSP70 can be released via both active secretion pathways and EVs, it is conceivable that HIP may follow similar routes; however, this remains entirely speculative and warrants direct investigation.

Future studies will be required to determine whether HIP co-immunoprecipitates with insulin and/or HSP70 in plasma, to define its binding stoichiometry, and to elucidate its cellular origin and release mechanisms under stress conditions.

### Translational and physiological considerations

#### Sympathetic contribution and alternative mechanisms

Another important aspect that warrants further investigation is the potential contribution of the sympathetic nervous system to the metabolic responses observed following HS. Thermal stress induces a hyperadrenergic state that may influence glucose metabolism through mechanisms such as hepatic glycogenolysis.^20^

Although such mechanisms may contribute to the transient hyperglycemia observed following HS, our current data are more consistent with an HSP70/HIP-dependent mechanism, as evidenced by the complete abrogation of glucose intolerance following anti-HSP70 monoclonal antibody treatment. This observation strongly supports a primary role for extracellular HSP70 in modulating insulin bioavailability under these conditions.

Nevertheless, we fully acknowledge that alternative mechanisms, including increased hepatic glucose production, should be formally evaluated. Future studies should assess glycemic responses in parallel with sympathetic activity and pharmacological adrenergic blockade, as well as directly evaluations of hepatic glucose metabolism, including glycogen content and glycogenolysis, gluconeogenic gene expression, and tracer-based metabolic flux analyses during HS in the presence and absence of glucose overload. Such approaches will be essential to determine the relative contribution of neuroendocrine versus chaperone-mediated mechanisms in the metabolic response to HS.

#### Post-translational modifications and other chaperones

The present study did not evaluate PTM) of HSP70, which may influence its interaction with client proteins. The potential role of PTMs in modulating extracellular HSP70–insulin binding remains to be determined. Moreover, we did not assess other HS proteins, such as HSP60 or HSP90,^107^ in plasma. Therefore, we cannot exclude the possibility that additional chaperones contribute to the extracellular stress response. Whether other HS proteins are capable of interacting with insulin or modulating hormone bioavailability, as well as their broader roles within the extracellular chaperone network, remains to be determined in future studies.

The present findings also raise the possibility that targeting extracellular HSP70 may represent a strategy to modulate metabolic inflammation and insulin availability. Experimental studies in animal models have shown that neutralization of extracellular HSP70 improves insulin resistance and metabolic dysfunction under high-fat diet conditions.^68^, ^77^ In this context, humanized anti-HSP70 monoclonal antibodies^78^ may offer a means to selectively target extracellular, pro-inflammatory HSP70 without interfering with its intracellular cytoprotective functions. However, the therapeutic relevance, safety, specificity, and long-term effects of such interventions remain to be established and should be regarded as hypothesis-generating. Additionally, the present study did not assess potential immunogenic responses associated with monoclonal antibody administration, nor downstream effects on glucose transporter regulation or insulin signaling pathways. These aspects should be addressed in future studies.

#### Chronic metabolic context

Finally, an important open question concerns the potential contribution of extracellular HSP70 to hyperinsulinemia and metabolic dysregulation in chronic inflammatory states. Given that elevated circulating HSP70 and insulin levels frequently coexist in conditions such as obesity and T2DM, it is conceivable that HSP70-mediated insulin sequestration contributes to the maintenance of compensatory hyperinsulinemia. In this framework, extracellular HSP70 may act as a previously unrecognized regulator of insulin bioavailability, linking stress responses, inflammation, and metabolic control. While this hypothesis remains speculative, it provides a mechanistic basis for future studies aimed at defining the role of extracellular chaperones in endocrine regulation. A schematic summary of these concepts is presented in Figure 6, depicting a temporal model of extracellular versus intracellular HSP70–insulin interactions.

## Materials and methods

### Materials - general

All common chemicals were purchased from Sigma-Aldrich (São Paulo, Brazil) unless otherwise stated.

Antibodies were from Sigma, Enzo Life Sciences (Farmingdale, NY, USA), Abcam Inc. (Boston, MA, USA), and Santa Cruz Biotechnology (Dallas, TX, USA). Details on validation, cross-reactivity, and titers are appropriately described when employed in specific methods (below).

Recombinant rat, mouse, and bovine proteins were from Enzo Life Sciences.

PBS (Sigma) consisted of 136.8 mM NaCl, 2.7 mM KCl, 0.9 mM KH_2_PO_4_, and 6.4 mM Na_2_HPO_4_, pH 7.4.

BSA is fraction V bovine serum albumin (Sigma, A2153).

HBSS (Hanks’ Balanced Salt Solution, Sigma) consisted of 137.0 mM NaCl, 3.0 mM KCl, 0.3 mM Na_2_HPO_4_, 0.5 mM KH_2_PO_4_, 0.8 mM Na_2_SO_4_, 1.3 mM CaCl_2_.2H_2_O, 0.8 mM MgCl_2_.6H_2_O, 4.2 mM NaHCO_3_, pH 7.4. For *ex vivo* studies with muscle slices, HBSS was equilibrated with 5 % CO_2_/95 % O_2_ (v/v) carbogen mixture, pH 7.4.

Freshly prepared protease and phosphatase inhibitor cocktail (Sigma) consisted of (final concentrations) 4.2 μM leupeptin, 0.31 μM aprotinin, 20 μM TLCK (N-tosyl-l-lysine chloromethyl ketone, hydrochloride), 100 μM PMSF (phenyl-methyl-sulfonyl fluoride), 1 mM Na_3_VO_4_, 1 mM Na_2_MoO_4_, and 1 mM β-glycerophosphate.

Foetal bovine serum (FBS) was heat-inactivated, endotoxin-free, sterile-filtered (0.1 μm) serum from Cripion Biotecnologia, Andradina SP, Brazil (catalogue no. FB0010SI).

Prior to protein reactions, both autologous rat serum and FBS were dialyzed (cutoff 12-16 kDa) twice against 100 volumes of PBS, pH 7.4, at 4 °C for 12 h and sterilized by filtration (0.22 μm membranes, Millipore, USA) as previously described,^108^ having protein contents assessed.^109^

Radioactive material was supplied by PerkinElmer Inc. (Waltham, MA, USA). ^14^C-glucose (catalogue no. NEC042V250UC) is d-[^14^C(U)]-Glucose (300 mCi/mmol), ^14^C-2DG (catalogue no. NEC720A250UC) is 2-deoxy-d-[^14^C(U)]-glucose (310 mCi/mmol) and ^3^H-2DG (catalogue no. NET328001MC) is 2-[1,2-^3^H (N)]-deoxy-d-glucose (8 Ci/mmol). The disposal of radioactive waste was according to the rules of the Brazilian National Nuclear Energy Commission (CNEN, licenses no. AP-0745 and 18179) that align with the International Atomic Energy Agency Safety Standards.

### Ethical compliance

The investigation followed all ethical rules established by Arouca’s Act (Brazilian Federal Law no. 11794/2008) and the 8th Edition of Guide for Care and Use of Experimental Animals published by National Research Council of the National Academies (2011; available at https://grants.nih.gov/grants/olaw/guide-for-the-care-and-use-of-laboratory-animals.pdf) in accordance with ARRIVE Guidelines 2.0 (Available https://www.nc3rs.org.uk/arrive-guidelines, accessed 28 April 2023) and the NIH’s Principles and guidelines for reporting preclinical research (Available from www.nih.gov/research-training/rigor-reproducibility/principles-guidelines-reporting-preclinical-research, accessed 16 May 2023. All the procedures (protocols no. 19858, 21082, 21354, 23451, and 23811) were reviewed and approved by the Committee of Animal Welfare of the Federal University of Rio Grande do Sul (CEUA-UFRGS), which adheres to guidelines of The Brazilian National Council for the Control of Animal Experimentation (CONCEA).

### Animals

Adult (60 days old) male Wistar rats inbred at The Federal University of Rio Grande do Sul Institute of Basic Health Sciences Animal Care Facility (CREAL) were randomly assigned (https://www.graphpad.com/quickcalcs/randomN2/) and used. The animals were maintained under controlled temperature (23 °C ± 1 °C) and humidity (60 %) in a 12/12 h light/dark cycle (lights on at 07:00 am), having free access to water and pelleted rodent standard chow (NUVILAB CR-1, Nuvital Nutrients S.A., Curitiba, Brazil) comprising total metabolizable energy of 16.6 MJ/kg, being 11.4 % as fats, 62.8 % as carbohydrates, and 25.8 % as proteins. The rats were housed in autoclavable polypropylene cages (49 × 34 × 16 cm; internal area of 1430 cm^2^), with 4 animals per cage. Sample size for each experiment is given in the figure legends. The animals, evaluated by the attending veterinarian, were in excellent health and showed no signs of infection during the studies. They were also test-naïve.

### *In vivo* heat shock protocol

Before the experiments, the rats were fasted (or fed *ad libitum*) overnight for 12 h from 08:00 pm to 08:00 am, when the HS protocol started. HS employed herein is a hot-tubbing protocol adapted from previous works.^13^, ^21^, ^110^ Briefly, the animals were anesthetized (i.p.) with a mixture of 90 mg.kg^−1^ ketamine and 10 mg.kg^−1^ xylazine comprising a total volume of 140 μL/100 g of body weight, and used 10–15 min later, as soon as anesthesia was ensured by the loss of foot and corneal reflexes. After the induction of anesthesia, the animals were immersed in a water bath (42 °C) up to the shoulder with the help of a rubber tie to avoid drowning. Core temperature was monitored with a rectal thermometer (Minipa MT-455A; electrode dimensions: 8 ×2 mm, in length and diameter, respectively) throughout the period in which the animals were kept in the thermal bath. After reaching a rectal temperature of 41 °C, the animals remained in the bath, with continuous temperature monitoring (41 °C-41.7 °C) for 15 min. Control animals (sham-treated) were anesthetized but maintained at room temperature, being warmed with the help of a heating pad and infra-red device to maintain core temperature between 36.5 °C and 37.5 °C for the same time period. Room temperature during HS (or sham) sessions was settled at 27 °C. After the HS (or sham) sessions, the animals were rehydrated with a subcutaneous (interscapular) injection of sterile PBS (20 mL/kg of body weight) and allowed to recover from anesthesia under infrared warming until reaching 37 °C (or to maintain 37 °C in the case of sham group), as they remain with no automatic thermal regulation during anesthesia. Afterwards, the animals were kept in the animal facility until the other procedures (glycemic tests, blood biochemistry, or euthanasia for blood and tissue collection) to be carried out.

It is important to highlight that the relatively short HS exposure (41.5 °C-42.0 °C for 15 min) was deliberately chosen to induce a controlled and transient stress sufficient to promote the release of pre-existing HSP70 into the extracellular compartment, rather than to maximize *de novo* HSP70 synthesis. This approach is consistent with established protocols used in previous in vivo studies from our group and others.^13^, ^21^, ^54^, ^59^, ^110^

Of note, longer HS durations (e.g., 60–120 min) are well known to robustly activate the HSR, leading to significant transcriptional upregulation and protein synthesis of HSP70. While such conditions are appropriate for studying intracellular chaperone induction, they are less suitable for investigating the rapid mobilization and extracellular release of preformed HSP70 pools, which was the primary focus of the present study.

In support of this interpretation, pharmacological inhibition of HSP70 synthesis using quercetin did not alter the hyperglycemic response observed 12 h after HS (Fig 3**c**), indicating that the effects described are largely independent of *de novo* HSP70 production and are more consistent with the mobilization of pre-existing HSP70. Previous work from our group has also demonstrated that prolonged HS (e.g., 2 h at 42 °C) is required to achieve maximal endogenous HSP70 synthesis,^4^ further supporting the distinction between these experimental paradigms.

### Glucose and insulin tolerance tests

Intraperitoneal glucose tolerance (ipGTT;^111^) and intraperitoneal insulin tolerance (ipITT;^11^, ^112^) tests were performed either immediately after HS sessions (time zero) or after 6, 12, or 24 h after HS (or sham) sessions. Before glucose or insulin administration, the animals were allowed to rest for at least 30 min (time zero) in the laboratory. Blood samples were collected from the tail tip with the help of an Accu-Chek Fast Clix lancing device kit (Roche, São Paulo, SP) adjusted for the minimal depth (setting = 0.5), yielding a 0.5 μL drop of blood for the tests, which used Optium Xceed Glucometer (Abbott Brasil, São Paulo, SP, Brazil). Blood was collected just before (time zero) and at 30, 60, 90, and 120 min after glucose overload (1 g/kg body weight) for ipGTT or just before (time zero) and at 15, 30, and 45 min after 1.0 UI/kg insulin (Humalog Lispro insulin, Eli Lilly do Brasil, São Paulo, SP, Brazil) administration for ipITT. Glucose and insulin solutions were prepared in PBS, and the procedures were always carried out in the morning (approximately 09:00 h) with a 2-week interval between ipGTT and ipITT tests in the same animal. To calculate the incremental areas under the curves (iAUC) of ipGTT and the inverted incremental areas under the ipITT curves (inv-iAUC), the trapezoid quadrature method by the similarity of triangles (from Thales’ theorem^113^) was used.^114^, ^115^, ^116^

### Estimation of glycemic status and the appearance of glucose intolerance

We used both the Homeostasis Model Assessment-Insulin Resistance (HOMA-IR)^117^ and the Quantitative Insulin Sensitivity Check Index (QUICKI)^118^ to estimate glycemic status during the experimental maneuvers. Although HOMA-IR has been commonly utilized as an estimate of glycemic status in humans, numerous studies have been conducted to validate its applicability in both rats and mice. Notably, investigations into insulin sensitivity during pregnancy in Wistar and Sprague-Dawley rats,^119^ cardiovascular risk biomarkers and diabetic cardiomyopathy in insulin-resistant streptozotocin (STZ)-T2DM rats,^120^ β-cell dysfunction in female Sprague-Dawley rats,^121^ and a dedicated study validating HOMA-IR in a high-fat diet (HFD)-induced insulin-resistant model in Wistar rats^122^ have revealed that HOMA-IR is a valid measure to determine insulin resistance in Wistar rats exhibiting similarities between human and rat models.

HOMA-IR values were calculated using the equation: [Fasting Plasma Insulin (μU/mL) × Fasting Plasma Glucose (mmol/L)] / 22.5. QUICKI was determined by the equation: QUICKI = 1/[log(I_0_) + log(G_0_)], with I_0_ representing fasting insulin (μU/mL) and G_0_ being fasting glycemia (mg/dL). A HOMA-IR score above 2.5 was assumed as an indicative of insulin resistance, akin to humans, while QUICKI values between 0.450 and 0.339 denote normal glycemic status, and values below 0.339 suggest insulin resistance.

Insulinogenic index (IGI) was calculated during ipGTT tests by the formula ΔI_30_/ΔG_30_, where ΔI_30_ and ΔG_30_ are, respectively, the variations of insulin and glucose concentrations from time zero to the point 30 min during ipGTT.^123^, ^124^

For the conversion of insulin to SI units, we assumed 5807.57 g/mol,^125^ so that 1 μU = 5.975 fmol e 1 μU/mL = 5.975 pmol/L (≈ 6 pM).

### HSPA1A, HSPA8, HIP, and antibody injections

In order to mimic the *in vivo* HS effects observed during the 30-min peaks of glycemia during ipGTTs, animals were submitted to the same protocol of ipGTT but, instead of HS, 12-h fasted rats were injected (i.v.) with LPS-free HSP72 (hspa1a) or HSP73 (hspa8) 12 h before the ipGTTs. The doses of chaperones were calculated on the basis of maximal median HSP70 and insulin values attained at the 30-min peaks observed during ipGTTs (HS+glucose point in the figures), as follows: 139 pM HSP72, 139 pM HSP73 and 565 pM insulin. Therefore, 5 nM HSP72/HSP73 was used to simulate 10 times this maximal insulin concentration found. The same rationale was used in relation to HIP injection, i.e., to be equimolar with HSP72 and HSP73 (139 pM or 5 nM). When injected in combination, the proteins were not mixed to be administered simultaneously. Instead, the proteins were administered in successive and individual injections into the animals. The amount of the proteins to be administered was calculated to attain the above plasma concentrations on the basis of the following formula for the estimated rat blood volume (BV) with respect to its body weight: BV = [(0.06 × body weight) + 0.77] mL of blood.^126^ Animals were injected with *ca*. 10 μL of these solutions into the lateral tail vein using a 23 G x 1.6 mm needle in an insulin syringe.

### Recombinant proteins injected

HSP72/hspa1 (low-endotoxin, mouse, recombinant hspa1a = 98.2 % amino acid homology with human HSPA1A/HSPA1B) = Enzo ADI-ESP-502 (ATPase activity assay positive).

HSP72/HSPA1 (low-endotoxin, human, recombinant, Enzo ADI-ESP-555 (ATPase activity assay positive).

HSC70/hsp73 (low-endotoxin, bovine, recombinant hspa8) = ADI-SPP-751 (ATPase activity assay positive).

HIP (rat, recombinant) = Enzo ADI-SPP-767 (54 kDa).

### Antibodies injected

In order to check whether the *in vivo* HS effects during the 30-min peaks of glycemia during ipGTT could be abolished by anti-HSP70 administration, the animals were submitted to the same protocol of ipGTT but were injected (i.p., **∼**35 μL of 0.2 mg/mL solution per kg of rat body weight) with anti-HSP70 mAb (clone 3A3) antibodies 2 h before the HS. Clone 3A3 mAb recognizes hspa1a, hspa1b and hspa8 forms of HSP70. The doses of antibodies were calculated on the basis of estimated BV = [(0.06 × body weight) + 0.77] mL of blood as a function of body weight^126^ and considering maximal median HSP70 values attained at the 30-min peaks observed during ipGTT (HS+glucose point in the figures), namely 139 pM HSP72 and 139 pM HSP73 (approximately 10 ng/mL). Then, the molar number of binding sites for IgG molecules (assuming 150,000 μg/μmol) per volume of antibody (at 0.2 mg/mL) of 2.667 pmol/μL was calculated to equal the estimated HSP70 concentrations. We assumed that i.p. mAb equilibrate with extracellular fluid and plasma in less than 4 h and that the presence of mAb in the blood persists for more than 24 h.^127^, ^128^

### Euthanasia

The animals (fasted or fed *ad libitum*, as properly described in the figure legends) were killed by decapitation. Euthanasia always occurred in a separate laboratory environment with exhaustion to avoid the influence of fear pheromones^21^ and was conducted by an experienced researcher. Between the death of an animal and another, the guillotine and the rest of the material were completely sanitized with water, detergent, and alcohol. Considering the need to obtain peripheral blood for the analysis of HSR components, animal killing under anesthesia, while desirable, is incompatible with the experimental goals because all anesthetics commonly employed with experimental animals lead to intense and sustained hyperglycemia in rodents.^129^, ^130^, ^131^ Moreover, these anesthetics interfere with the function of cells involved in the production of HSP72, such as leukocytes.^132^ Considering also that plasma concentrations of HSP70, which is a major molecular object of the study, is greatly suppressed by high levels of plasma glucose,^10^ animal killing was performed without anesthesia. In experiments in which the animals were anesthetized for HS protocol and killed soon after (time zero), basal glycaemia typically rose from 4.7 ± 0.3 to 10.3 ± 0.7 mM (mean ± s.d.) due to anesthesia. The method chosen for euthanasia is based on the fact that it is an effective method that produces minimal physiological changes in the tissues, considering the need to carry out cellular and biochemical analyses. Once euthanasia was carried out, the collection of blood and tissues was initiated immediately.

### Blood biochemistry

Following the manufacturer’s instructions, triacylglycerols (triglycerides) were assessed in plasma samples by using the Invitrogen Triglyceride (TG) Colorimetric Assay Kit (Catalogue no. EEA028). Circulating FFA were assayed in the plasmas (EDTA) treated with freshly prepared protease/phosphatase inhibitor cocktail and analyzed by EnzyChrom™ Free Fatty Acid Assay Kit (BioAssay Systems, Hayward, CA, USA). Lactate was measured during blood collection alongside glucose and insulin tolerance tests with the Accutrend Plus Kit MG/DL (Roche, catalogue no. 0017-0016).

### Glucose and insulin measurements

In addition to ipGTT and ipITT, analyses of fasting glycemia and fasting insulinemia were also performed in blood samples collected at the time of euthanasia in heparinized tubes, containing (final concentrations) 2.5 IU/mL heparin (Hepamax, Blau Farmacêutica, Cotia, SP, Brazil) and 3 mg/mL sodium fluoride to block glycolysis. Plasma samples obtained after centrifugation of whole blood for 15 min at 1600 x *g* under refrigeration (4 °C) were then submitted to glucose analysis with LabTest (Labtest Diagnóstica S.A., Lagoa Santa, MG, Brazil) colorimetric test (#133).

Insulin was measured using rat insulin competitive EIA kit (Cayman # 589501, commercialized by Bertin Pharma, France, SPI-Bio #A5105; which 100 % cross-reacts with rat, mouse, human, hamster, porcine, and sheep insulin). For insulin assays, plasmas were treated with (final concentrations) 6.5 μg/mL *o*-phenantroline and 22.6 μg/mL EDTA to avoid any interference of possible hemolysis, as per the manufacturer’s instructions. Insulin detection limit was 0.08 ng/mL and the assay range was 0.08-10.00 ng/mL; interassay CV: 6.5 %-8.5 %; intra-assay CV: 0.7 %-2.5 %.

### Plasma HSP72 and HSP73 measurements

Plasma (2 mg/mL disodium EDTA) samples destined to the quantitation of the 72 kDa (hspa1a and hspa1b) and 73 kDa (Hsc70/hspa8) members of the HSP70 chaperone family were treated (as per manufacturers’ instructions) with freshly prepared protease and phosphatase inhibitor cocktail (Methods-General section).

HSP72 assays were conducted using the AMP’D® HSP70 High Sensitivity hspa1a-specific (0.016 % cross-reactivity with HSP73/hspa8) sandwich ELISA kit (catalogue no. ENZ-KIT-101, Enzo Life Sciences). HSP72 limit of detection under the manufacturer’s conditions was 7 pg/mL (0.097 pM), intra-assay precision is 7.0 % for 1.92 ng/mL (27.7 pM) and 15.0 % for 140 pg/mL (1.95 pM), whereas inter-assay precision is 10 % for 1.85 ng/mL (25.7 pM) and 7.7 % for 140 pg/mL (1.95 pM). To further ensure the robustness of our measurements, we compared the AMP’D® assay with an independent high-sensitivity ELISA (Enzo Life Sciences; catalogue no. ADI-EKS-715), obtaining comparable results, with the AMP’D® kit providing a lower limit of detection.

HSP73 was analyzed in plasma samples by the sandwich hspa8-specific (no cross-reactivity with hspa1, hspb1, or hspd1) ELISA Kit (StressMarq Biosciences Inc., Victoria, BC, Canada, catalogue n° SKT-106). HSP73 limit of detection under the manufacturer’s conditions was 1.54 ng/mL (21.1 pM), intra-assay precision is 8.0 %, whereas inter-assay precision is 9.0 %.

As previously reported,^133^ there is a good correlation between values obtained from hspa1a/hspa1b and hspa8-specific kits, with values not significantly different between kits, although the ENZ-KIT-101 (AMP’D® HSP70 kit, the same used in our work) demonstrated excellent reproducibility between individual assay kits, with duplicate measurements highly correlated between assays (R2 = 0.99). Hspa8-specific ELISA kit SKT-106 has also been previously validated in samples of NAFLD/MASH patients, demonstrating high correlation with HSPA8 SNPs.^134^

We note that different analytical approaches may preferentially detect distinct extracellular pools of HSP70. For example, membrane-associated HSP70 on EVs or cell surfaces can be assessed using specific antibodies such as cmHsp70.1.^34^ While such approaches may provide complementary information regarding vesicular or membrane-bound HSP70, they fall outside the scope of the present study, which focused on quantifying circulating HSP70 isoforms in plasma.

### Co-immunoprecipitations and protein separation

For co-immunoprecipitation assays,^135^, ^136^, ^137^, ^138^, ^139^ plasmas (EDTA) from rats (treated under different experimental conditions) were diluted (1:4, by volume) with non-denaturing immunoprecipitation (IP) buffer,^135^ consisting of 150 mM NaCl, 1 % (v/v) Nonidet P-40 % and 0.5 % (w/v) sodium deoxycholate in 10 mM Tris-HCl pH 7.4. Then, 500 μL of the diluted plasma were pre-cleared with 10 μL of BSA-pre-blocked Protein A/G Plus-Agarose resin (Santa Cruz Biotechnology, sc-2003) for 90 min at 4 °C under agitation in a platform rocker. Afterwards, the samples were centrifuged (15,000 x *g* for 30 s) and the supernatants (*ca*. 500 μL) were saved to form immune complexes with 5 μL (2 μg) of guinea pig anti-insulin whole serum (Sigma, I8510, 100 % of cross-reactivity with insulin of rat, mouse, and human origin) for 12 h at 4 °C under agitation. Alternatively, immune complexes (2 μg) were prepared using mouse monoclonal antibody for HSP70 (Sigma H5147). Please see details on the antibodies used in immunoprecipitations in the next section.

After Protein A/G Plus-agarose pre-clearing and before the addition of antibodies for the immunological reactions and immunoprecipitations, the incubation media were pre-cleared with 2 μg/mL of non-immune mouse IgG (Sigma, NI03) from normal mouse serum (in immunoprecipitations of plasmas for the detection of insulin or HSP70) or with anti-HSP70 antibody (2 μg/mL) when the samples were intended to competition between cold and biotin-labeled insulin. After pre-clearing procedures, the samples were immunoprecipitated with 10 μL of BSA-pre-blocked Protein A/G Plus-Agarose resin for 90 min at 4 °C under agitation.

After immune reactions, 10 μL of Protein A/G Plus-Agarose beads were added to the samples, which were further incubated for 90 min at 4 °C under agitation. Next, samples were centrifuged (15,000 x *g* for 30 s) at room temperature (22 °C-25 °C) and the beads were washed 3 times (15,000 x *g* for 30 s) with 1 mL of IP buffer and once with 1 mL of PBS pH 7.4. Target proteins were eluted from the beads by direct dispersion in 55 μL of non-reducing th 0.3 % (w/v) Red Ponceau S (Sigma) in Laemmli’s sample buffer containing 2.0 % (w/v) SDS, 10 % (v/v) glycerol, 0.05 % (w/v) bromophenol blue in 62.5 mM Tris-HCl pH 6.8 to be heated (95 °C) for 5 min being processed for gradient polyacrylamide electrophoresis (4 % stacking gel, 5 %-20 % resolving gel, in terms of total acrylamide/bis-acrylamide) minigels for 4 h at 15 mA/gel (*ca*. 40 μg protein/well). Running buffer (no SDS) was 25 mM Trizma base, 192 mM glycine, pH 8.3. Afterwards, proteins were transferred onto nitrocellulose membranes (GE HealthCare) according to the electrotransfer (Bio-Rad) manufacturer’s instructions (2 h, 100 V) and transferred bands were visualized with 0.3 % (w/v) Red Ponceau S (Sigma) in 3 % (w/v) TCA solution to be photodocumented (ImageQuant 350, GE HealthCare).

To study the *in vivo* interactions of HSP70 and insulin, we used plasmas from rats (either fed or fasted for 6 or 15 h) treated with 1 IU/kg body weight of long-acting myristoyl insulin Determir (Levemir, Novo Nordisk Brasil) or fast-acting Lispro insulin (Humalog Lispro, Eli Lilly do Brasil) as described in the figure legends. Afterwards, plasma samples were immunoprecipitated for insulin (and immunoblotted for HSP70) or immunoprecipitated for HSP70 (and immunoblotted for insulin).

To give further insight into the binding of HSP70 to insulin, equimolar amounts (139 pM, equivalent to 10 ng/mL HSP70 in plasma) of both proteins (mouse hspa1a and Humalog Lispro insulin) were incubated for 90 min at 37 °C under agitation in 100 μL (total volume) of Hanks’ balanced salt solution (HBSS). The reaction was stopped with 400 μL of ice-cold IP buffer and submitted to IP for 12 h, as described above, under two different conditions: IP for insulin followed by immunoblot for HSP70 and IP for HSP70 followed by immunoblot for insulin. Appropriate controls were run. Antibodies against insulin and HSP70 were the same as referred to above. After IP, samples were eluted from the beads by the dispersion in 55 μL of native (non-reducing, no SDS) sample buffer containing 2 % (v/v) glycerol, 0.01 % (w/v) bromophenol blue in 60 mM Tris-HCl pH 6.8. Samples were not heated and were processed by native discontinuous gradient electrophoresis (4 % stacking gel, 10 %-20 % resolving gel) in minigels as described below (running buffer: 25 mM Trizma base, 192 mM glycine, pH 8.3).

As the above experiments with incubations of insulin and HSP70 in HBSS **did not** replicate the *in vivo* binding observed through the IP of plasmas (above), we repeated the incubations of equimolar amounts (139 pM) of insulin and HSP70 in dialyzed serum or whole plasma (obtained from 12 h fasted rats) for 90 min at 37 °C under agitation in a total volume of 100 μL. Prior to reactions, sera and plasmas were dialyzed twice as stated in the Methods-General section. Then, samples were immunoprecipitated and processed for native electrophoresis as described here. For the immunodetection of insulin, guinea pig anti-insulin whole serum (Sigma I8510, at 1:1000 dilution) followed by goat anti-guinea pig IgG (whole molecule)-HRP labeled (Sigma A7289, at 1:10,000 dilution) were employed. For the immunodetection of HSP70, mouse mAb (Sigma H5147, at 1:1000 dilution), followed by rabbit anti-mouse IgG (Sigma A9044, 1:10,000) was used. For certain loading controls, we used biotinylated goat anti-mouse IgG Fc-specific (Sigma, B7401, at 1:80,000 dilution), which recognizes both guinea pig and mouse IgG Fc.

For competition studies, the samples (125 μL final volume) were incubated in dialyzed (Methods-General section) FBS or dialyzed rat serum (from 12-h fasted animals) and increasing concentrations of Lispro insulin whose final concentrations in the test were 0, 0.69, 1.38, 2.76, 5.50, 11.04, and 69 μM (the equivalent of 0, 0.5, 1.0, 2.0, 4.0, 8.0, and 50 µg of protein), in the presence of 1.38 μM of biotinylated insulin (Sigma, I2258, 1.1 mol biotin/mol insulin from bovine pancreas) and 1.38 μM HSP70 (mouse haspa1a), for a period of 2 h at 37 °C in a water bath with shaking. Afterwards, samples were immunoprecipitated for HSP70 and submitted to immunoblotting with streptavidin-HRP. For Kd studies, 1.38 μM HSP70 (mouse hspa1a) was incubated in the presence of increasing concentrations of biotin-insulin (from 10^−13^ to 10^−5^ M) in dialyzed rat serum for 2 h at 37 °C, under the same above conditions, to be immunoprecipitated for HSP70 and immunoblotting with streptavidin-HRP. HSP70 concentration used (1.38 μM) is equimolar with 1 mg/mL insulin-biotin (172.2 pmol/125 μL in the assay). For the negative control assays (only dialyzed FBS or dialyzed rat serum), insulin and HSP70 were omitted, and the samples were immunoprecipitated with 2 μg/mL of non-immune mouse IgG from normal mouse serum (Sigma, NI03). For the positive control assays, insulin was only added to dialyzed FBS or rat serum to be immunoprecipitated for HSP70.

For the detection of HSP70 (hspa1a, hspa1b, and hspa8), HIP, HSP40 (DnaJA1), HSP20 (hspb6), BAG-1, and HspBP1 by immunoblotting in plasma samples, rats were fasted (or fed *ad libitum*) overnight for 12 h and submitted to an HS (or sham) session as described above. Then, the animals were euthanized, and blood collected in disodium EDTA (2 mg/mL) to be treated with freshly prepared protease and phosphatase inhibitor cocktail (above). Afterwards, equal amounts of protein (*ca*. 40 μg; assessed by the Pierce™ BCA Protein Assay Kit, catalogue no. 23225, Thermo Scientific, Waltham, MA, USA), were processed by conventional SDS-PAGE and electrotransfer onto nitrocellulose membranes to be processed by immunoblotting (below) for either HSP70 (Sigma H5147 mouse mAb, 1:1000, followed by rabbit anti-mouse IgG, Sigma A9044, 1:10,000), HIP (rabbit mAb, Enzo, ADI-SPA-766, 1:1000, followed by goat anti-rabbit IgG HRP-labeled, Sigma A4914, 1:10,000), DnaJA1 [rabbit polyclonal (pAb), Enzo, ADI-SPA-400, 1:1,1000, followed by goat anti-rabbit IgG HRP-labeled, Sigma A4914, 1:10,000), Hspb6 (rabbit pAb, ENZO, ADI-SPA-796-D, 1:1000, followed by goat anti-rabbit IgG HRP-labeled, Sigma A4914, 1:10,000), BAG-1 (rabbit pAb, Enzo, ALX-210–009-R050, anti-mouse/rat Bcl-2 associated athanogene-1, molecular chaperone regulator, 1:1000, followed by goat anti-rabbit IgG HRP-labeled, Sigma A4914, 1:10,000) or HspBP1 (rabbit pAb, Invitrogen PA535155, 1:1000, followed by goat anti-rabbit IgG HRP-labeled, Sigma A4914, 1:10,000). Plasma albumin was used for gel loading control.

For immunoblotting procedures, SNAP i.d. (Merck Millipore) quick immunoblot vacuum system was used.^4^ The membranes were washed with water, blocked in 1 % (w/v) BSA in wash buffer [50 mM Tris, 5 mM EDTA, 150 mM NaCl (TEN) Tween 20 (0.1 % w/v) solution, pH 7.4] for 30 s, and then washed three times (30 s each) with wash buffer. Subsequently, the membranes were incubated for 10 min with mouse anti-human HSP70 monoclonal antibody (Sigma H5147, clone BRM-22 ascites fluid), recognizing both the 73 kDa ‘constitutive/cognate’ (*hspa8* gene product) and the 72 kDa ‘inducible’ (*hspa1a* and *hspa1b* gene products) forms of the HSP70 of human, mouse, and rat origin, at a 1:5000 dilution (by volume) in wash buffer. After three washes with wash buffer (30 s each), membranes were probed with horseradish peroxidase (HRP)-labeled secondary antibody (rabbit anti-mouse IgG whole molecule, Sigma A9044, 1:10,000) for 10 min. Membranes were then washed three times (30 s each) with wash buffer and protein detection was performed by the enhanced chemiluminescence method. For the detection of HRP-labeled reagents, ECL Prime (GE HealthCare, cat. no. GERPN2232) was used following the manufacturer’s instructions. As gel loading controls in IP, IgG whole molecules were used (see details below). For plasma immunoblot assays, we used albumin as a gel loading control, while for the detection of insulin receptors in muscle cells, β-actin (anti-β-actin mAb, HRP-labeled - Sigma, A3854), at 1:5000 dilution) was the loading control.

After chemiluminescence reactions, protein bands were photodocumented for 600 s (60 frames, 1 photo every 10 s) and quantified in ImageQuant™ 350 chemiluminescence system (GE HealthCare) and the accompanying online stacking imaging software ImageQuant TL 7.0.

### Description of antibodies, recombinant proteins and immunoreagents

#### Primary antibodies

**Anti-HSP70**: Mouse anti-human HSP70 monoclonal antibody (Sigma, H5147, clone BRM-22 ascites fluid), at 1:5000 dilution. This antibody recognizes both the 73 kDa “constitutive/cognate” (*hspa8* gene product) and the 72 kDa “inducible” (*hspa1a* and *hspa1b* gene products) forms of the HSP70 of human, mouse, and rat origin.

For *in vivo* injection of anti-HSP70 antibodies, we used mouse anti-HSP70 mAb (Santa Cruz, sc-32239, clone 3A3, raised against human HSPA1A/B and HSPA8), which recognizes HSP70 of human, mouse and rat origin.

Alternatively, the 72 kDa inducible HSP70 was investigated by using mouse mAb clone C92F3A-5 (Enzo, ADI-SPA-810 unlabeled IgG_1_ and ADI-SPA-810FI FITC-labeled IgG1) which only and specifically recognizes the *hspa1a* and *hspa1b* gene products of human, rat, mouse, rabbit, sheep, guinea pig, and *Drosophila sp.*, at 1:1000 dilution.

**Anti-Insulin**: Guinea pig anti-insulin, whole serum (Sigma, I8510), which recognizes insulin of human, rat, mouse, pig, and bovine origin, at 1:1000 dilution.

**Anti-HIP**: Rabbit mAb (Enzo, ADI-SPA-766), at 1:1000 dilution.

**Anti-DnaJA1**: Rabbit pAb (Enzo, ADI-SPA-400), which recognizes DnaJA1 (HSP40) of human, mouse, rat, and non-human primate origin, at 1:1,1000 dilution.

**Anti-HspBP1**: Rabbit pAb (Invitrogen PA5–35155), which recognizes HspBP1 of human, rat, mouse, and origin, at 1:1000 dilution.

**Anti-HSP20**: Rabbit pAb (ENZO, ADI-SPA-796-D), which recognizes HSP20 (hspb6) of human, rat, and mouse origin, at 1:1000 dilution.

**Anti-BAG-1**: Rabbit pAb (Enzo, ALX-210–009-R050), anti-mouse/rat Bcl-2-associated athanogene-1, BAG family molecular chaperone regulator 1, at 1:1000 dilution.

**Anti-Insulin receptor** (∼350 kDa): Rabbit pAb, which recognizes a 95 kDa fragment of mouse, rat, and human IR in SDS-PAGE (Sigma, SAB4501555), at 1:500 dilution.

**Anti-Phosphorylated** (Tyr972) insulin receptor (∼350 kDa): Rabbit pAb (Sigma, I1783), which recognizes a 95 kDa fragment of human and mouse phospho-IR in SDS-PAGE, at 1:500 dilution.

### Horseradish peroxidase (HRP)-labeled antibodies

Rabbit anti-mouse IgG (whole molecule), HRP-labeled (Sigma, A9044), at 1:10,000 dilution.

Goat anti-guinea pig IgG (whole molecule), HRP-labeled (Sigma, A7289), at 1:10,000 dilution.

Goat anti-rabbit IgG (whole molecule), HRP-labeled (Sigma, A4914), at 1:10,000 dilution.

Mouse anti-β-actin mAb, HRP-labeled (Sigma, A3854), at 1:5000 dilution.

### Biotin-labeled reagents

Biotin-insulin (from the bovine pancreas) conjugate was from Sigma (I2258), 1.1 mol biotin/mol insulin. In competition studies, 1.38 μM of both insulin-biotin and HSP72/hspa1 (Enzo, either ADI-ESP-502 mouse recombinant or ADI-ESP-555 human recombinant) with increasing concentrations of cold insulin were used. For Scatchard plots intended to Kd calculations, we used 1.38 μM HSP72/hspa1 (Enzo, either ADI-ESP-502 mouse recombinant or ADI-ESP-555 human recombinant) and increasing concentrations of biotin-insulin (from 10^−13^ to 10^−5^ M).

Goat anti-mouse IgG (Fc-specific), biotin-labeled (Sigma, B7401), at 1:80,000 dilution. This antibody recognizes both guinea pig and mouse IgG Fc.

Rabbit anti-goat IgG (whole molecule), biotin-labeled (Sigma, B7014), at 1:200,000 dilution

Goat anti-rabbit IgG (whole molecule), biotin-labeled (Sigma, B8895) at 1:80,000 dilution

Ultrasensitive streptavidin-peroxidase Polymer (Sigma, S2438) was employed to detect biotin-labeled reagents at 1:200 dilution.

All the antibodies were validated by immunoblotting using ten-fold serial dilutions of authentic standards of each protein of interest (from 1.39 nM to 1.39 pM). Also, antibodies were tested by increasing dilutions, starting with the minimum dilution suggested by the manufacturers up to the highest dilution that still permitted protein visualization by ECL. Cross-reactivity and specificity were tested using unrelated proteins.

### *In vivo* uptake of radiolabeled 2-deoxyglucose (2-DG) and incorporation of radiolabeled glucose

To assess the effects of HS on glucose metabolism, the rats were fasted overnight for 12 h from 08:00 pm to 08:00 am, when the HS protocol started. Twelve hours after HS, the animals were submitted to an ipGTT, as described above, except that glucose overload was prepared with [^3^H]-2DG in 0.8 g/mL cold glucose (1 g/kg body weight) for glucose uptake studies and with [^14^C]-glucose in 0.8 g/mL cold glucose (1 g/kg body weight) for incorporation studies. Detailed specifications on the radiochemicals are described above (Methods-General section). Each animal received 0.157 µCi of either radiotracer per 100 g of body weight (*ca*. 0.47 μCi/rat). The animals were then euthanized 40 min after glucose overload (uptake) or after 60 min (incorporations). In another set of experiments, instead of being submitted to HS, the animals were injected with recombinant mouse/rat LPS-free HSP72 (hspa1a) and tested by radiolabeled ipGTT 12 h later. Simultaneously to blood collection, the following tissues were surgically excised and freeze-clamped (liquid N_2_) by another researcher: whole heart, visceral adipose tissue (epididymal and retroperitoneal), brown adipose tissue, soleus and gastrocnemius muscles, whole encephalon, hypothalamus, kidney, jejunum, ileum, colon, whole pancreas, liver, circulating lymphocytes, spleen, thymus, and circulating erythrocytes. Blood was collected in heparinized tubes (2.5 IU/mL of blood), and erythrocyte mash was washed 3 times with cold PBS. Each tissue was weighed and homogenized in water (5 mL/g tissue) still frozen. When dealing with [^14^C]-glucose incorporations, the tissues were homogenized in the presence of NaF (4 mg/g of tissue) to avoid glucose metabolism during the preparation. Hemolysis of erythrocyte mash was conducted in water containing 4 mg/mL NaF. The hearts were exsanguinated and washed with PBS, and the intestines were also cleaned with PBS before being homogenized. After homogenization, samples were pelleted (16,000 x *g* for 1 min at 4 °C) and dissolved in Ultima Gold scintillation cocktail (PerkinElmer) in 2 mL polyethylene scintillation vials (1.8 mL final volume) for the radioactivity (dpm) of the supernatant fractions to be counted in a liquid scintillation counter (PerkinElmer) and converted into μmol/100 g tissue (or μmol/mL of pelleted erythrocytes) of 2-DG taken up or glucose incorporated.

### *Ex vivo* uptake of radiolabeled 2-deoxyglucose (2-DG) by soleus muscle

To evaluate the effects of HSP70 on insulin-stimulated glucose uptake in skeletal muscle, we incubated longitudinal slices of the soleus, gastrocnemius, and rectus abdominis muscles from normal, 12-h-fasted rats. However, since this was an *ex vivo* incubation without perfusion, the size of the muscles to be used was a decisive factor in the choice of model. Therefore, we used soleus muscle strips (4 samples per animal) surgically excised from 12 h fasted rats and equilibrated the muscles for 30 min in modified Warburg flasks (with central glass well with a capacity of 500 μL) under agitation (60 rpm) at 37 °C in HBSS equilibrated with 5 % CO_2_/95 % O_2_ (v/v) carbogen mixture, pH 7.4. After the equilibration phase, the muscle strips were incubated at 37 °C for additional 30 min in the presence or absence of 10 μU/mL insulin (60 pM, so equimolar with 4.3 ng/mL HSP72) in HBBS containing (or not) dialyzed autologous serum (we tested 0 %, 5 % and 10 % by volume and chose 10 % in the subsequent experiments), 1 mM ADP (disodium salt, Sigma), and increasing concentrations of recombinant rat/mouse LPS-free HSP72 (hspa1a at 0, 5, 10, 20, 100, or 500 ng/mL, which is equivalent to 0, 69.5, 139, 278, 1390, and 6950 pM, respectively) under the same above conditions. The experiments started with the addition of [^3^H]-2DG (0.47 μCi/mL) in either 2.75, 5.5, or 11 mM cold glucose. ADP was added (or not, in controls) to the incubation media considering that, during the cycle of anchoring and release of client proteins at the protein/substrate binding site (SBD) of the HSP72 chaperone, ATP promotes the release of the peptides, and ADP maintains them in the active site. For this reason, in studies of HSP70 binding to client proteins, ADP is used.^26^ Incubations with test substances at 37 °C for 30 min after a 30 min equilibration period was defined in pilot experiments, which indicated that the maximum plateau of 2-DG uptake is attained under these experimental conditions. After incubations, muscle strips were freeze-clamped (liquid N_2_), weighed, and homogenized in water (10 mL/g) to be processed for liquid scintillation as described above. In another set of experiments, the rats were fasted overnight for 12 h from 08:00 pm to 08:00 am, when the HS protocol started. Twelve hours after HS (or sham treatment), the animals were killed, and soleus muscles surgically excised and equilibrated for 30 min in HBSS containing 0.1 % (w/v) BSA at 37 °C. Afterwards, tissues were incubated in the presence of 11 mM glucose and a pharmacological dose of Humalog Lispro insulin (10 mU/mL) for 2 min at 37 °C, without addition of HSP72, under agitation to be immediately frozen and processed for SDS-PAGE and immunoblotting for total and Tyr972-phosphorylated forms of insulin receptors.

### *In vitro* uptake of radiolabeled 2-deoxyglucose (2-DG) by cultured L6 cells

Rat myoblast L6 cells (American Type Culture Collection, ATCC cat. no. CRL-1458) were cultured in RPMI 1640 culture medium (Sigma, R1383), containing 2 mM l-glutamine, 24 mM NaHCO_3_, 25 mM HEPES, 11 mM d-glucose, 100 U/mL penicillin, 100 μg/mL streptomycin) and 10 % (v/v) FBS, pH 7.4, in 24-well plates (500 μL final volume) at 37 °C in a 5 % (v/v) CO_2_ humidified atmosphere in air. To differentiate, cells were allowed to reach confluence, and the culture media was changed to that containing 2 % (vol/vol) FBS for 7 days, changing the medium every second day.^140^ Experiments were restricted to cells from passages 2-15, and undifferentiated cells were not allowed to grow more than 65 % confluence. Glucose uptake was measured using the 2-DG method described in^140^ with minor modifications. Briefly, cells were serum-starved overnight before each experiment, and glucose uptake was measured on day 7. In the morning of the experiment, the medium was replaced with a serum-free medium containing no glucose for 20 min. Then, the cells were exposed to increasing concentrations of recombinant rat/mouse LPS-free HSP72 (hspa1a at 0, 5, 10, 20, 100, or 500 ng/mL, which is equivalent to 0, 69.5, 139, 278, 1390 and 6950 pM, respectively) in RPMI 1640 medium containing 4.5 ×10^−3^ μCi/mL [^3^H]-2DG in 5.5 mM glucose, in the presence or absence of 10 μU/mL insulin as well as in the presence or absence of dialyzed FBS (10 % by volume) for 15 min at 37 °C. We tested 0 %, 2 %, 5 % and 10 % (v/v) dialyzed FBS and 10 % FBS was found to account for the best concentration for glucose uptake by the cells. ADP was omitted from the preparations. After the 15 min incubations, the dishes were placed in an ice bath and the cells washed twice with 1 mL of ice-cold PBS to be digested in 400 μL of 0.2 M NaOH for 1 h at 60 °C. Then, 200 μL of samples were transferred to Ultima Gold scintillation cocktail (PerkinElmer) in 2 mL polyethylene scintillation vials (1.8 mL final volume) and thoroughly mixed before being counted in a liquid scintillation counter (PerkinElmer).

### Construction of a molecular model of HSP70 (HSPA1A) and demonstration of its *in silico* interaction with insulin

#### Molecular modeling and docking

The first molecular model of HSP70 was constructed using the human amino acid sequence (GenBank Accession: AAA02807; Version: AAA02807.1; GenInfo Identifier: 292160). Templates from the Protein Data Bank (PDB), specifically entries #3C7N (HSP110:HSC70 Nucleotide Exchange Complex) and #3DPO (substrate binding domain of *E. coli* DnaK),^25^, ^141^ were employed, and MODELLER v9.8 software^142^ generated the.pdb files. Among fifty models, the optimal one was selected based on PDBsum server parameters (http://www.ebi.ac.uk/pdbsum/) and PROCHECK (EMBL-EBI, Cambridgeshire, UK) plots.^143^, ^144^ This HSP70 model includes the nucleotide-binding domain (NBD), SBD, and C-terminal domain.

To explore the interaction between HSP70 and insulin, PDB entry #1ZEI was used for insulin,^145^ and the SBD from our model was utilized in molecular docking experiments. These experiments were performed using the ClusPro 2.0 docking server (http://cluspro.bu.edu/).^146^, ^147^, ^148^, ^149^ Molecular graphics and analyses were conducted with the UCSF Chimera package (http://www.cgl.ucsf.edu/chimera).^150^ Chimera is developed by the Resource for Biocomputing, Visualization, and Informatics at the University of California, San Francisco, supported by grants from the National Institutes of Health National Centre for Research Resources (2P41RR001081) and the National Institute of General Medical Sciences (9P41GM103311).

#### Molecular dynamics calculations

The optimal docking solution for the insulin-HSP70/SBD (or PBD) complex, along with the free HSP70 system, was subjected to MD simulation using the GROMACS 4 suite (https://www.gromacs.org/)^151^ and the GROMOS96 (https://www.gromos.net/) 53a1 force field.^152^ The systems were solvated in triclinic boxes with periodic boundary conditions and SPC water models,^153^ and counterions were added to neutralize the system. The MD protocol followed methodologies established in previous studies.^154^, ^155^

The LINCS algorithm^156^ was employed to constrain covalent bond lengths, permitting an integration step of 2 fs following initial energy minimization via the Steepest Descents algorithm. Electrostatic interactions were computed using the Particle Mesh Ewald method.^157^ Temperature and pressure were maintained constant by coupling proteins, ions, and solvent to external baths with coupling constants of τ = 0.1 and 0.5 ps, respectively.^158^ The dielectric constant was set to ε = 1, and the reference temperature was 310 K. The system was gradually heated from 50 K to 310 K for progressive thermalization. The simulation proceeded for 75 ns without restraints. Hydrogen bonds were defined with a 3.5 Å distance between heavy atoms and a cutoff angle of 30° between hydrogen, donor, and acceptor atoms.^151^

Since the *in vivo* and *in vitro* experiments were carried out using rats, in a second round of *in silico* experiments, the structure of the hspa1a Nucleotide Binding Domain (NBD) from *R norvegicus* was modeled using the AlphaFold2 tool (https://alphafold.ebi.ac.uk/).^159^ The C-terminal portion, comprising residues 618–641, was removed as it did not present a defined structure, consisting of a long coil. As our *in vivo* studies showed that the HIP, but not other auxiliary chaperones, is present in the rat plasma, we investigated whether HIP could interact with the complex of rat hspa1 and insulin, thus altering the effectiveness of this interaction. Then, the middle domain of the HIP protein from *R norvegicus* was obtained from the PDB entry #4J8F,^23^ being employed in molecular docking calculations with the modeled structure of hspa1a as the target. For this purpose, the Lightdock server^160^ was used, and residues from the binding interface between the two proteins were used to guide the docking (based on the chimeric protein from PDB #4J8F) and calculated using the DIMPLOT program (EMBL-EBI, Cambridgeshire, UK) from the LigPlot+ suite,^161^ more specifically HIP domain residues 78, 79, 200, 239, 243, 245, and 246, and hspa1a residues 3, 141, 171, 215, and 382. Subsequently, insulin (PDB #1ZEI)^145^ was used as an input for a new molecular docking calculation against the formed hspa1a-HIP complex, with two main solutions being the highest ranked. Additionally, molecular docking of insulin alone was performed against the hspa1a model to evaluate the binding energy of the complex without HIP. The solutions found with these docking experiments had their binding energies calculated using the PyRosetta software (https://www.pyrosetta.org/),^162^ with 5 calculations for each case. The ΔG values of protein-protein interactions are given in Rosetta Energy Units (REU), which are now a stronger approximation of energies in units of kcal/mol.^24^

### Sample size

The sample size was calculated to detect the smallest expected difference between control and HS-treated animals (2 mM of rise in 30 min glycaemia during ipGTT^13^) assessed in 12 h fasted animals. A statistical power of 80 % was used for the calculation with a significance level of *P*< .05 and the DIMAM 1.0 Sample Dimensioning software for Windows from Editora Guanabara Koogan. This resulted in a minimum of 10 animals. The actual sample size for each test is given in the figure legends. Whenever experimentally and technically appropriate, the same animals were used for different analyses such as glycemia, insulinemia, HSP72 (hspa1a and hspa1b) and HSP73 (hspa8), lactate, triacylglycerols, and fatty acids. There was no exclusion of any animal, data, or samples during all the experiments. We report animal losses due to anesthesia-related fatalities, which is reflected in the uneven sample sizes shown in the figures.

### Randomization

The animals were randomly enrolled by simple randomization (https://www.graphpad.com/quickcalcs/randomN2/).

### Blinding

All the analyses were blindly performed by investigators unaware of the groups.

### Replication statement

For glucose tolerance tests carried out 12 h after HS treatment, the experiments (*n* = 4 each) were repeated 6 times with identical results, which were pooled and presented in the figures. Remaining groups (*n* = 3) were repeated twice. Experiments with radiolabeled tracers *in vivo* (*n* = 4 each) were repeated 3 times for HS treatment groups and twice in hspa1a injection groups. *Ex vivo* studies with isolated soleus muscle (*n* = 2 per round) were repeated 12 times for 11 mM groups, 4 times for 5.5 mM groups, and 3 times for 2.75 mM groups. *In vitro* experiments with L6 cells (*n* = 2 each) were repeated twice.

### Statistical analyses

Before the analyses, the outcome variables were assessed for normality through the Kolmogorov-Smirnov test. For the analyses of glycemic curves in ipGTT and ipITT, which are repeated-measures (RM) tests,^116^ a two-way RM analysis of variance (ANOVA) followed by Tukey’s multiple comparisons testing was used. Mixed-Effects analysis and Sidak multiple comparisons test was employed if values were missing. The areas under the glycemic curves for the comparison between control and HS groups were analyzed by the unpaired two-tailed Student’s *t*-test, while for the comparisons between 4 experimental groups in ipGTT and ipITT, 1-way ANOVA and Tukey’s multiple comparisons testing was employed. For IGI, data as medians and CI95 % were analyzed by the Kruskal-Wallis non-parametric ANOVA test, complemented by Dunn’s post-test. Inasmuch as various measurements of plasma HSP72 and HSP73 presented a high frequency of values equal to zero (below the limit of detection), a constant (0.5) was added to each of the data (x) for the ANOVA, so that, for the statistical analyses, ($\sqrt{x+0.5}$) was used as the input value to warrant homoscedasticity, as described.^163^ Transformed data were analyzed by 1-way ANOVA and Tukey’s multiple comparisons testing. However, the figures and tables show actual values (x). Individual adjusted *P*-values for each experiment were provided alongside data presentation and depicted in the figures when appropriate. The risk α for type I errors was settled for *P* < .05. GraphPad Prism 8.0.1 was used for statistical analyses and graphic representation. Data are presented as the means ± s.d.

## Ethics statement

The investigation followed all ethical rules established by Arouca’s Act (Brazilian Federal Law no. 11794/2008) and the 8th Edition of Guide for Care and Use of Experimental Animals published by National Research Council of the National Academies (2011; available at https://grants.nih.gov/grants/olaw/guide-for-the-care-and-use-of-laboratory-animals.pdf) in accordance with ARRIVE Guidelines 2.0 (Available https://www.nc3rs.org.uk/arrive-guidelines, accessed 28 April 2023) and the NIH’s Principles and guidelines for reporting preclinical research (Available from www.nih.gov/research-training/rigor-reproducibility/principles-guidelines-reporting-preclinical-research, accessed 16 May 2023. All the procedures (protocols no. 19858, 21082, 21354, 23451, and 23811) were reviewed and approved by the Committee of Animal Welfare of the Federal University of Rio Grande do Sul (CEUA-UFRGS), which adheres to guidelines of the Brazilian National Council for the Control of Animal Experimentation (CONCEA).

## Funding and support

This work has been supported by The Brazilian National Council for Scientific and Technological Development (CNPq, Grant no. 305822/2013–6, 303853/2017–4, and 305397/2021–4 to PIHBJ; Grant no. 563870/2010–9 to RC; Grant no. 307926/2022–2, 407329/2016–1, 307926/2022–2, 405546/2023–8, 444286/2024–1 and 403136/2024–5 to TGH; Grant no. 302959/2020–3 to MSK; Grant no. 427559/2018–9 to MSL) and The State of Rio Grande do Sul Foundation for Research Support (FAPERGS, Grant no. 19/2551–0001713–8 to PIHBJ; Grant no. 17/2551–0001424–3 to MSK). MSL, TGH, HTS, CHLM, AB, SPS, MAB, RRP, BBB, VSB, PMB, MILR, PRN were supported by fellowships from CNPq. Financial support from CAPES (Coordination of Superior Level Staff Improvement) is also acknowledged.

## Declaration of Generative AI and AI-Assisted Technologies in the Writing Process

During the preparation of this paper, the authors employed ChatGPT to improve conciseness, flow of ideas, and readability. After using the tool, the authors reviewed and edited the content as needed, taking full responsibility for the content of the publication.

## Declarations of interest

The authors declare that they have no known competing financial interests or personal relationships that could have appeared to influence the work reported in this paper.

## Data Availability

Data will be made available on request.

## Footnotes

Appendix A Supplementary data associated with this article can be found in the online at doi:10.1016/j.cstres.2026.100180.
