# Supplementary material for: Extracellular 70 kDa heat shock protein in blood plasma binds insulin and modulates glycaemic control in vivo
Source: Cell Stress Chaperones. 2026 May 10;31(4):100180. doi: 10.1016/j.cstres.2026.100180 (PMC13213666; doi:10.1016/j.cstres.2026.100180)
Supplement: Supplementary file 1 — Supplementary material [file mmc1.docx]

SUPPLEMENTARY FIGURES 1-11 and SUPPLEMENTARY TABLE 1


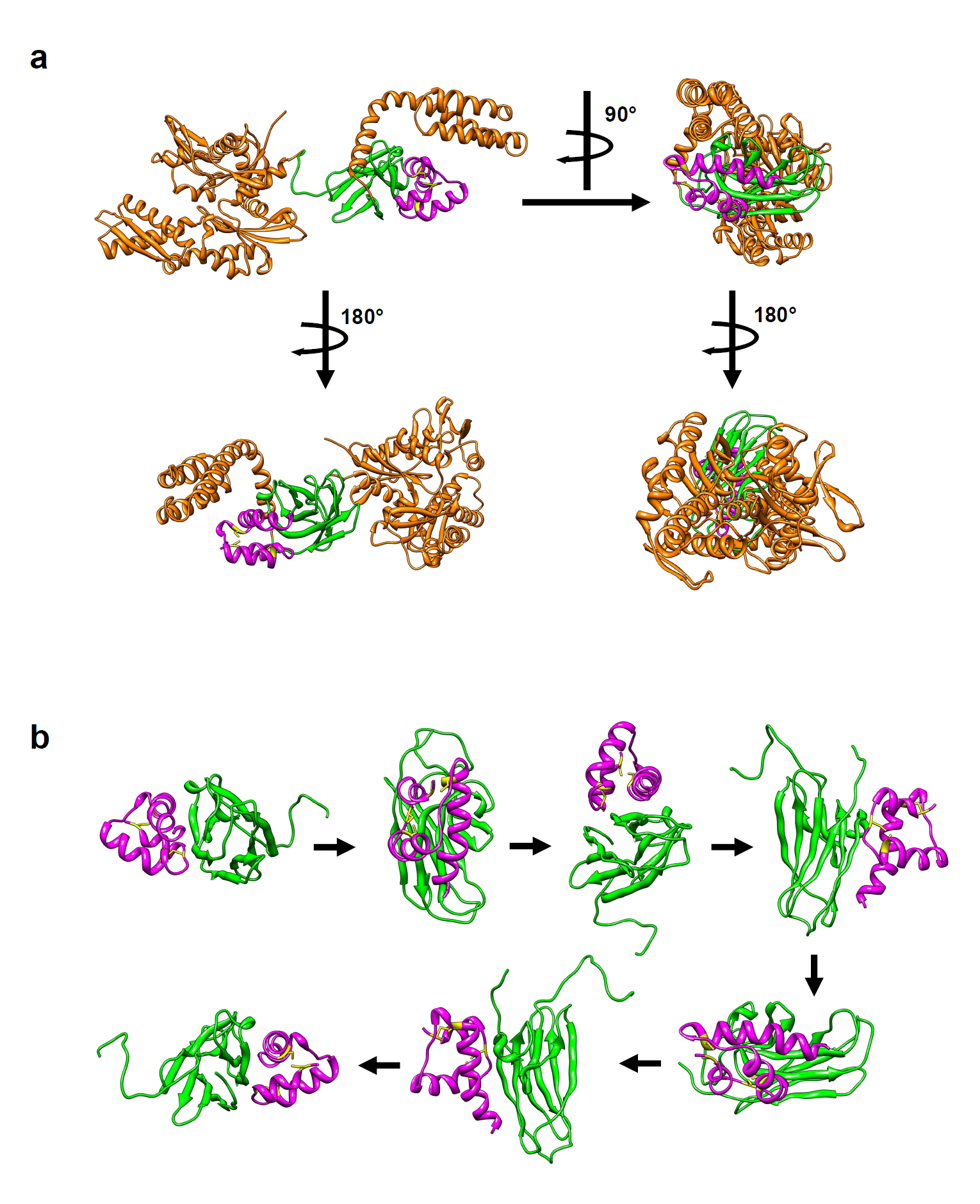
 **Supplementary Fig. 1. Rotational perspectives of HSP70-insulin docking.** **(a)** Different rotational perspectives of MD docking run between HSP70 (orange) and insulin (magenta). The HSP70 linker region is shown in green. **(b)** Different views of the trajectory of the MD run of the docking experiment result between HSP70 SBD (green) and insulin (magenta) from ClusPro 2.0 (http://http://cluspro.bu.edu/). For insulin, the disulfide-forming cysteines (Cys19 and Cys52, Cys43 and Cys38) are depicted in yellow.


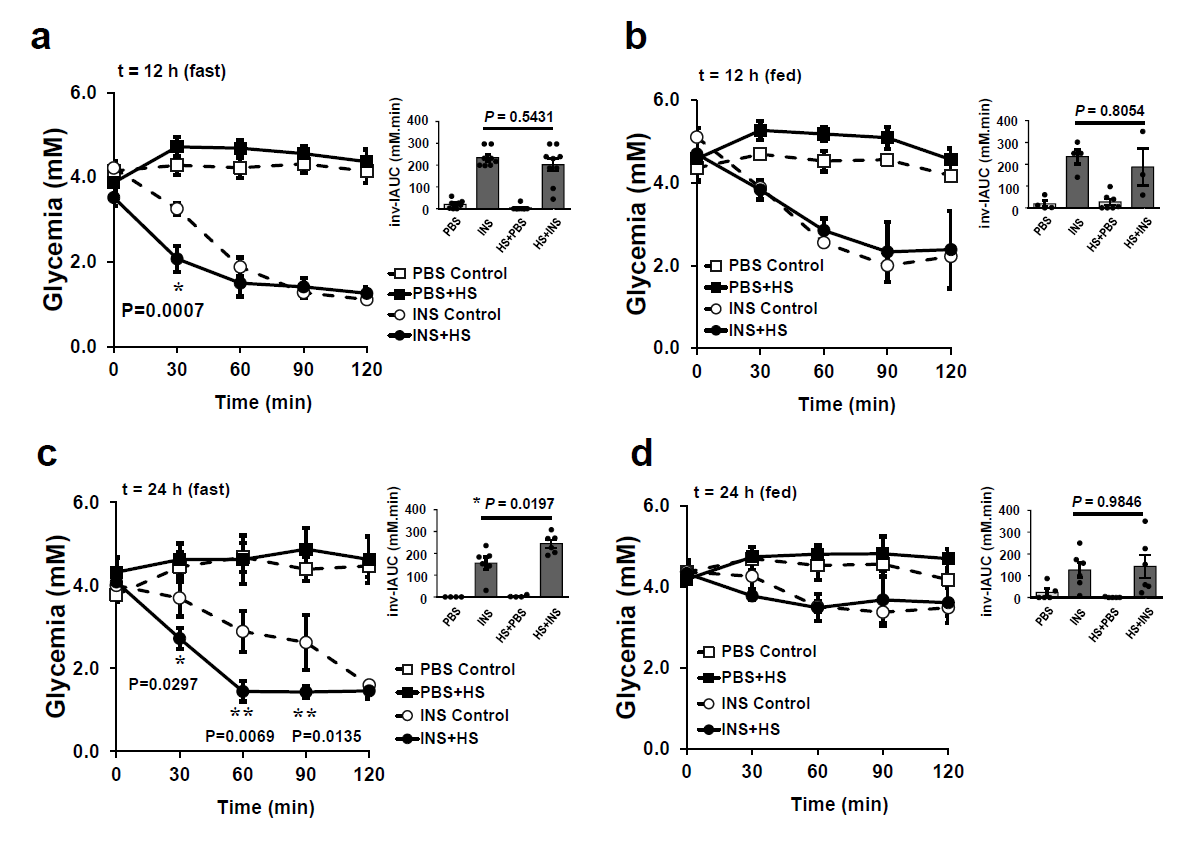
 **Supplementary Fig. 2. HS improves insulin sensitivity up to 24 h after HS treatment.** The complete set of experimental groups is shown. ipITT was carried out 12 or 24 h after heat treatment in 12 h fasted (**a** and **c**) or fed (**b** and **d**) rats. Differences in glycaemic curves were assessed by 2-way RM ANOVA followed by Tukey’s multiple comparison testing, whereas inv‑iAUC of ipITT were evaluated by 1-way ANOVA followed by Tukey’s multiple comparison testing, as described in the Methods section. Individual adjusted *P*‑values are given when appropriate. Data are the means ± s.d. (sample size is: **a)** *n* = 9, **b)** *n* = 4, **c)** *n* = 6, **d)** *n* = 6).


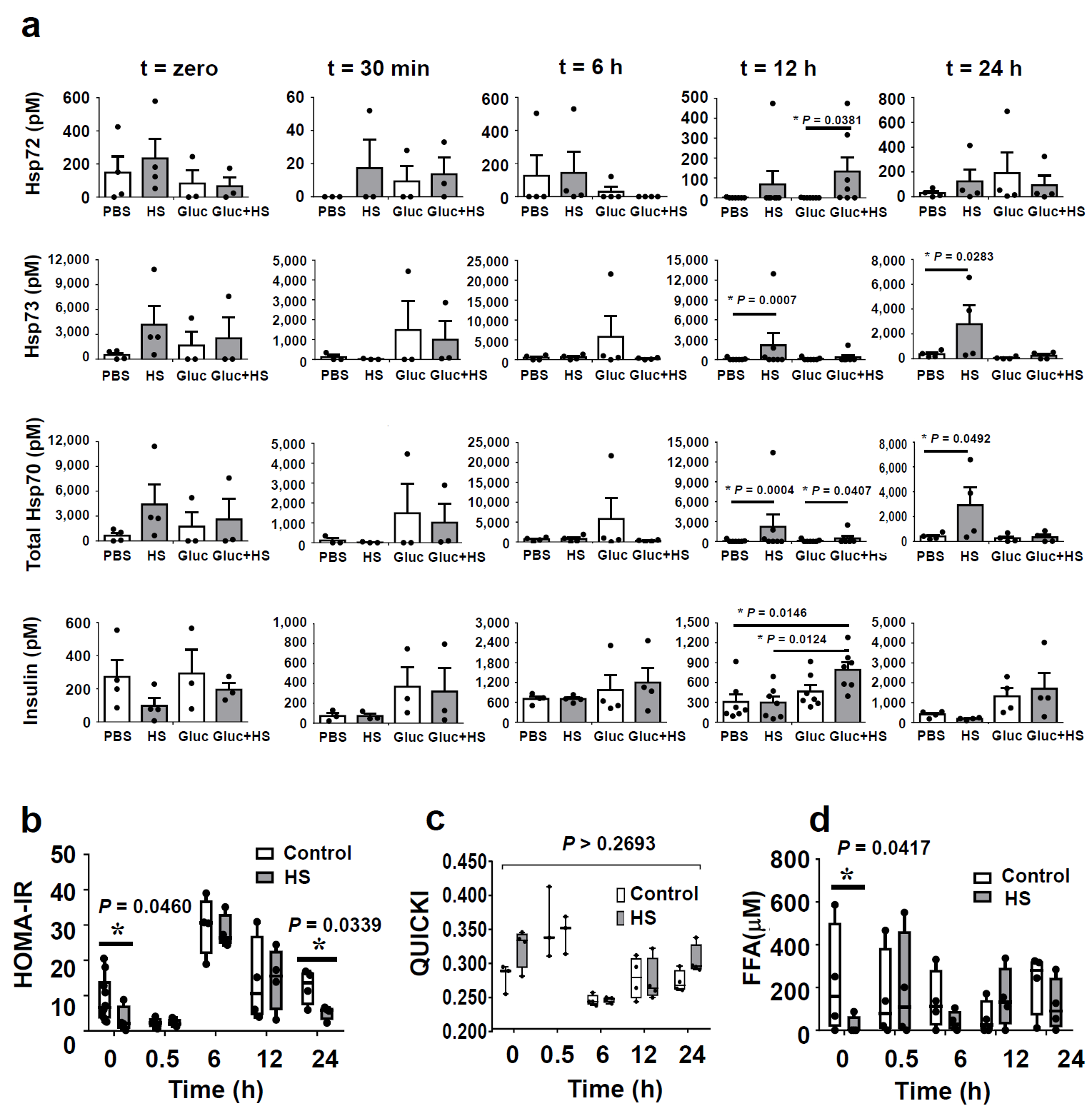


**Supplementary Fig. 3. Plasma concentrations of HSP70, insulin, free fatty acids and insulin resistance indices during GTT 12 h after HS. (a)** Plasma contents of HSP72 (hspa1a+hspa1b), HSP73 (hspa8), total HSP70 (=HSP72+HSP73) and insulin during ipGTT either just after HS (time zero, *n* = 4), or 30 min (*n* = 3), 6 h (*n* = 4), 12 h (*n* = 7) and 24 h (*n* = 4) after HS in 12 h fasted rats. Data are the means ± s.d. and were analysed by 1-way ANOVA followed by Tukey’s multiple comparison testing. **(b)** HOMA‑IR (*n* =4), **(c)** QUICKI (*n* = 4) and **(d)** plasma free fatty acids (FFA, *n* = 4) for the same time frame were evaluated by 2‑way ANOVA followed by the Tukey’s multiple comparisons testing with data displayed as box and whiskers including the medians and showing minimum to maximum values.


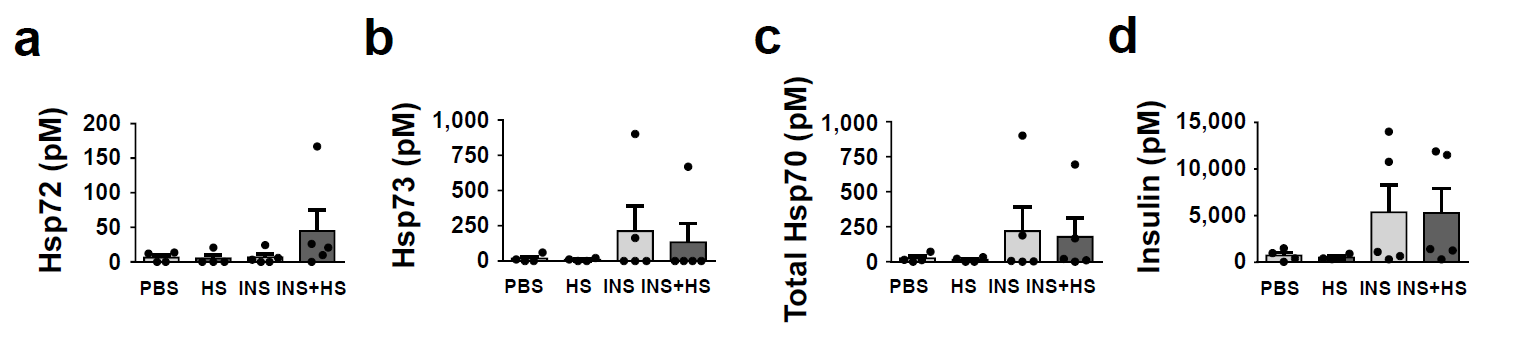
 **Supplementary Fig. 4. Plasma concentrations of HSP70 (HSP72 and HSP73) and insulin during ITT 24 h after HS.** Plasma contents of **(a)** HSP72 (hspa1a+hspa1b), **(b)** HSP73 (hspa8), **(c)** total HSP70 (=HSP72+HSP73) and **(d)** insulin at time 30 min after insulin injection during ipITT carried out 24 h after HS in 12 h fasted rats. Data were analysed by 1-way ANOVA followed by Tukey’s multiple comparison testing. Data are the means ± s.d.


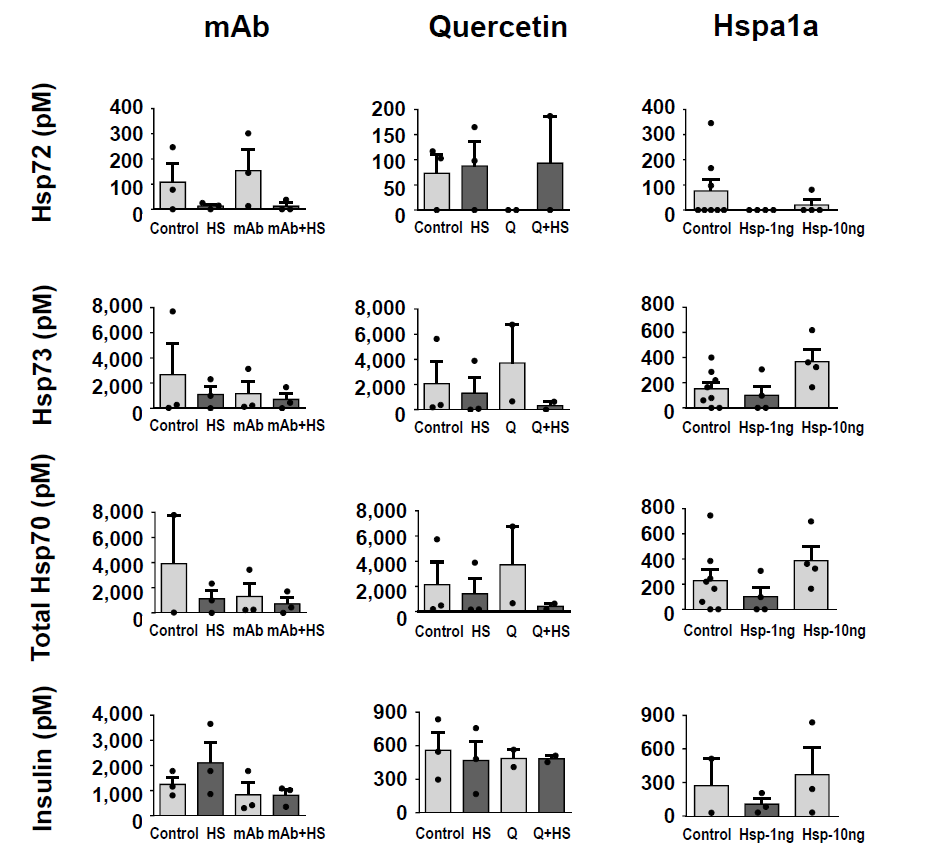
 **Supplementary Fig. 5. Effects of the administration of anti-HSP70 mAb, quercetin and Hspa1a on plasma concentrations of HSP70 and insulin during GTT 12 h after HS.** Plasma contents of HSP72 (hspa1a+hspa1b), HSP73 (hspa8), total HSP70 (=HSP72+HSP73) and insulin at time 30 min during ipGTT of 12 h fasted rats previously administered with either anti-HSP70 mAb, quercetin or hspa1a injections, as detailed in the Methods section. In the case of hspa1a injections, Hsp-1ng (or Hsp-10ng) denote that the chaperone was injected in an amount sufficient to attain either a 1 ng/mL (13.9 pM) or 10 ng/mL (139 pM) plasma concentration, respectively, as described in the Methods section. Data are the means ± s.d. and were evaluated by 1-way ANOVA followed by the Tukey’s multiple comparisons testing.


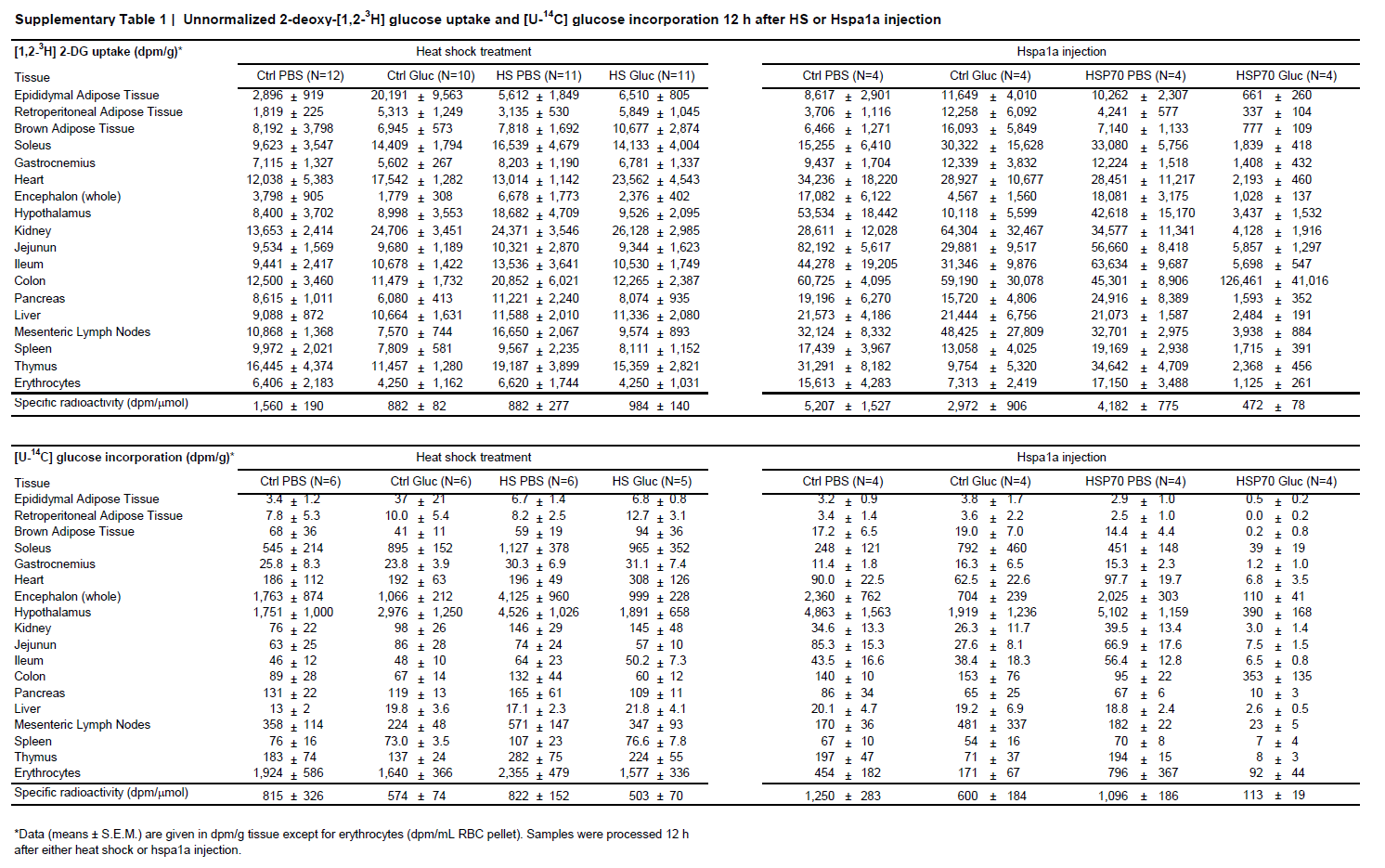


**Supplementary Tabel 1.** **Unnormalised [^3^H]-2DG uptake and of [^14^C]-glucose incorporations 12 h after HS or Hspa1a injections.** Unnormalised gross (dpm/g tissue) data for [^3^H]-2DG uptake and of [^14^C]-glucose incorporations are presented as the means ± s.d. and were analysed by 1-way ANOVA followed by Tukey’s multiple comparison testing. Sample size is the same as in Fig. 5 and are described in the Table headings.


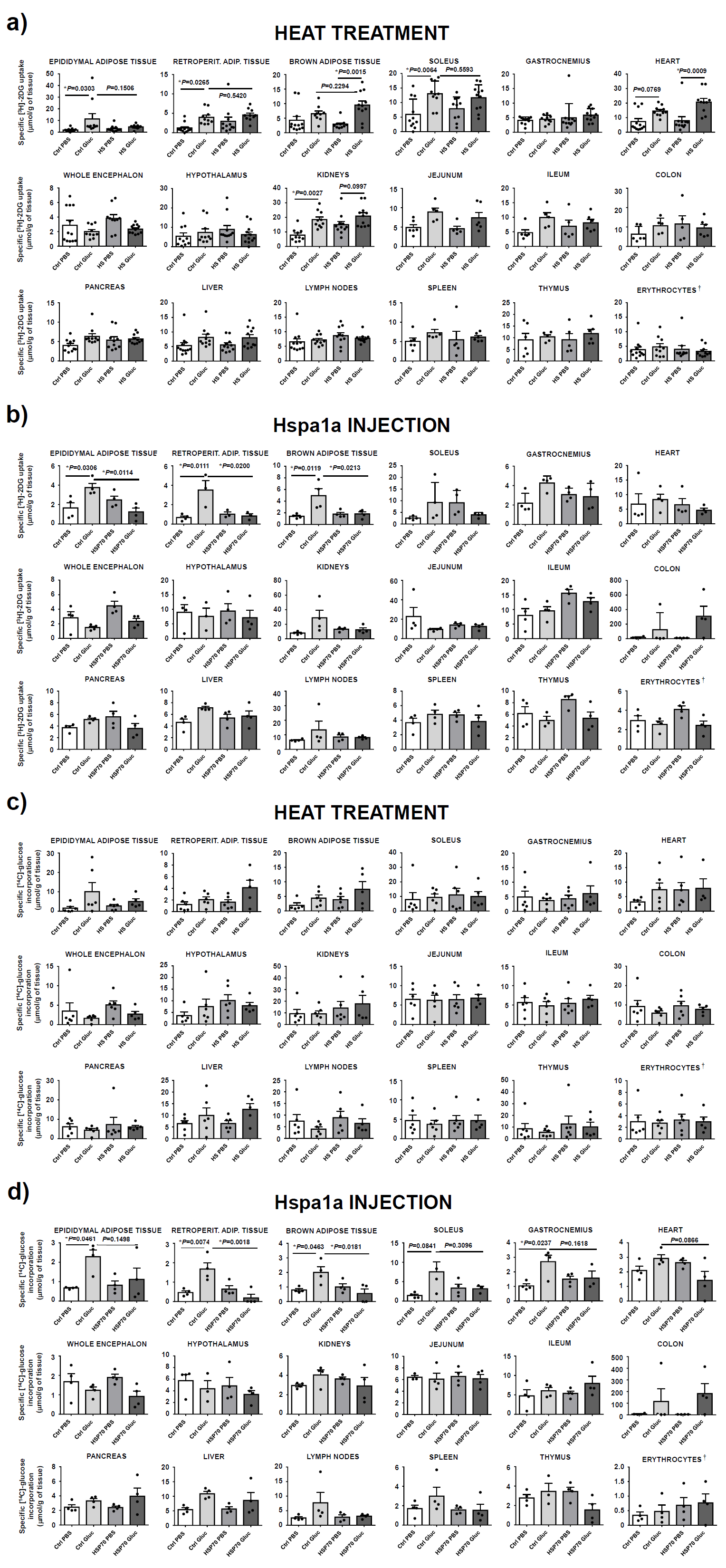


**Supplementary Fig. 6. Specific [^3^H]-2DG uptake and of [^14^C]-glucose incorporations 12 h after HS or Hspa1a injections.** Specific uptake of [^3^H]-2DG or [^14^C]-glucose incorporations (μmol/g tissue) are depicted as the means ± s.d. and were analysed by 1-way ANOVA followed by Tukey’s multiple comparison testing. Sample size is the same as in Fig. 5 and are described in the Supplementary Table 1 headings.


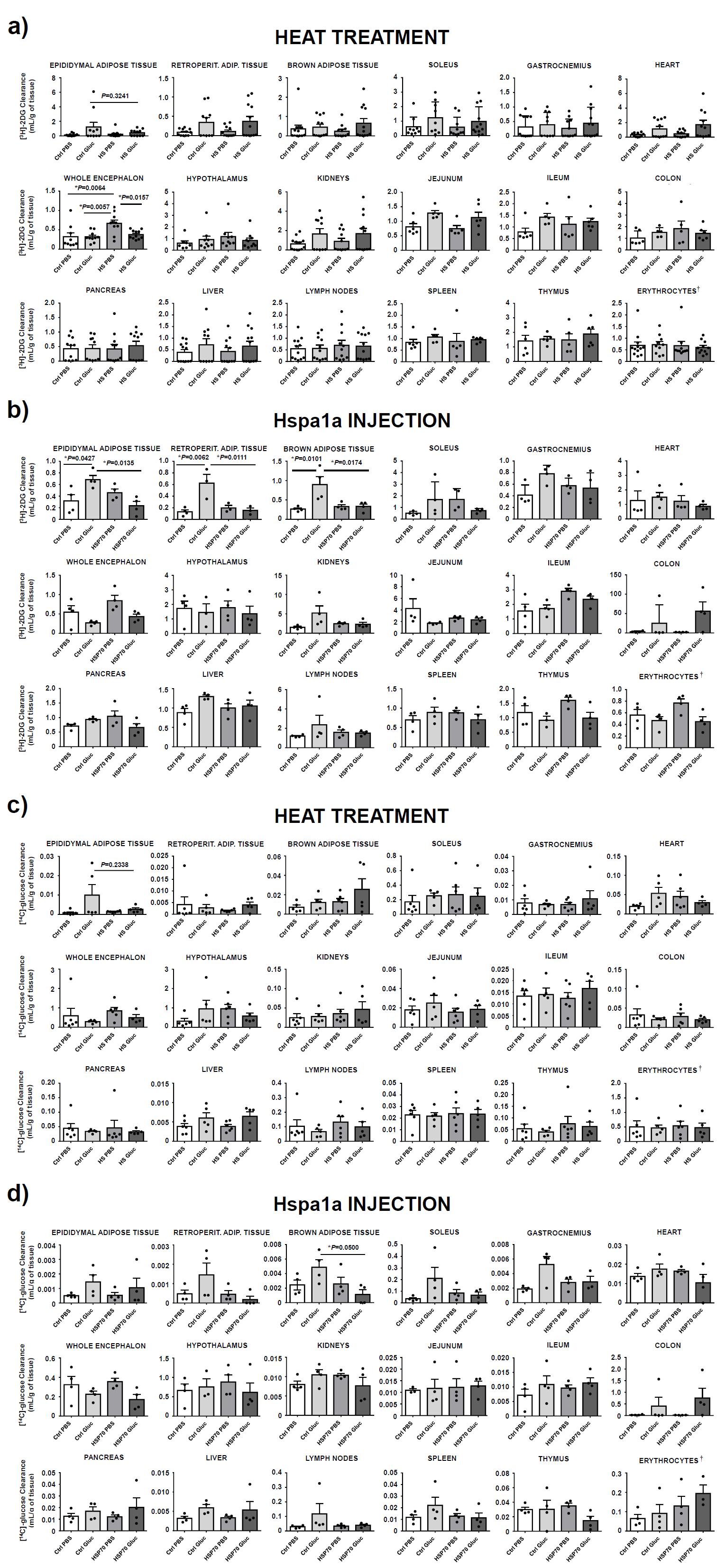


**Supplementary Fig. 7. Clearances of [^3^H]-2DG and [^14^C]-glucose 12 h after HS or Hspa1a injections.** Clearances (mL/g or mL/mL of RBC) for [^3^H]-2DG and [^14^C]-glucose are shown as the means ± s.d. and were analysed by 1-way ANOVA followed by Tukey’s multiple comparison testing. Sample size is the same as in Fig. 5 and are described in the Supplementary Table 1 headings.


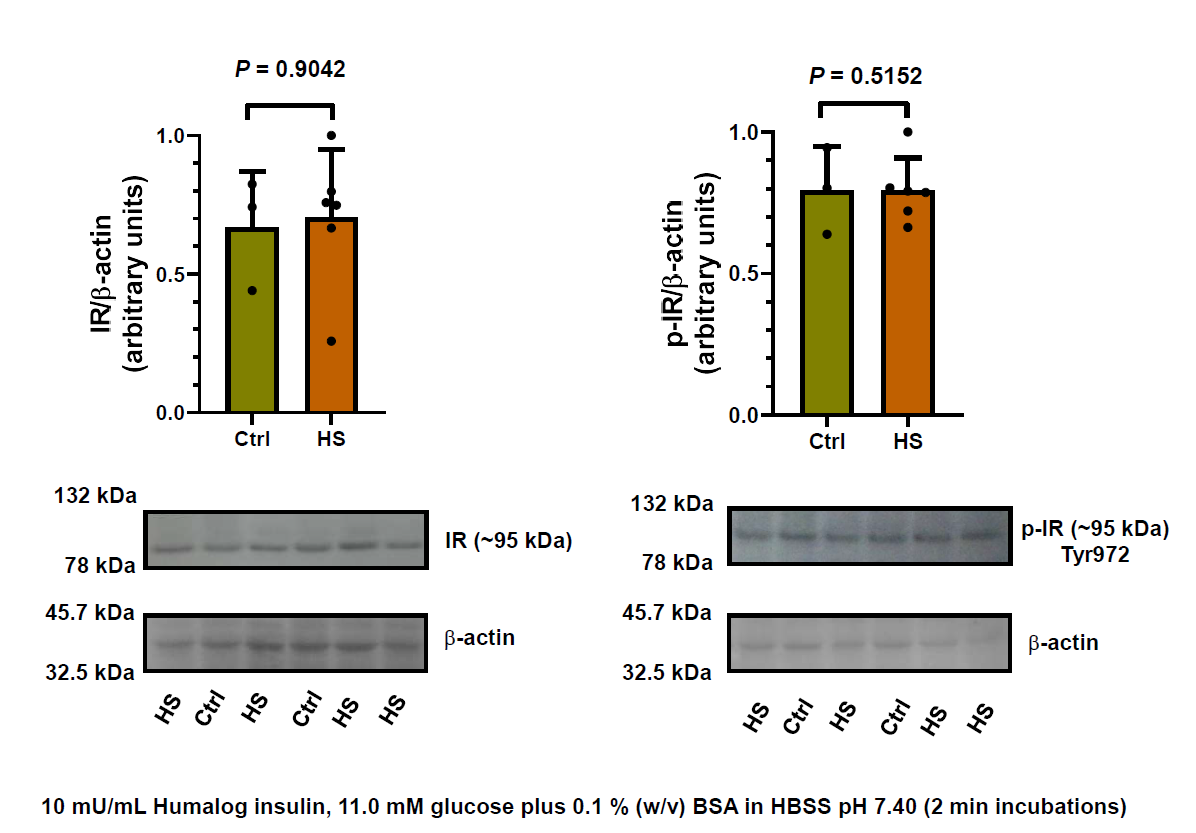


**Supplementary Fig. 8. HS treatment does not modify insulin receptor phosphorylation state in response to insulin in skeletal muscle.** Twelve-hour fasted rats were submitted to HS (or sham) treatment and, 12 h after HS (or sham treatment, Ctrl), the animals were killed and soleus muscles surgically excised and equilibrated for 30 min in HBSS containing 0.1 % (w/v) BSA at 37 °C. Afterwards, tissues were incubated in the presence of 11 mM glucose and a pharmacological dose of Lispro insulin (Humalog Lispro, 10 mU/mL) for 2 min at 37 °C, without addition of HSP72, under agitation to be immediately freeze-clamped (liquid N_2_) and processed for SDS-PAGE and immunoblotting for total and Tyr972-phosphorylated forms of insulin receptors. Data, expressed as the means ± s.d., were analysed by unpaired two-tailed Student’s *t*-test.


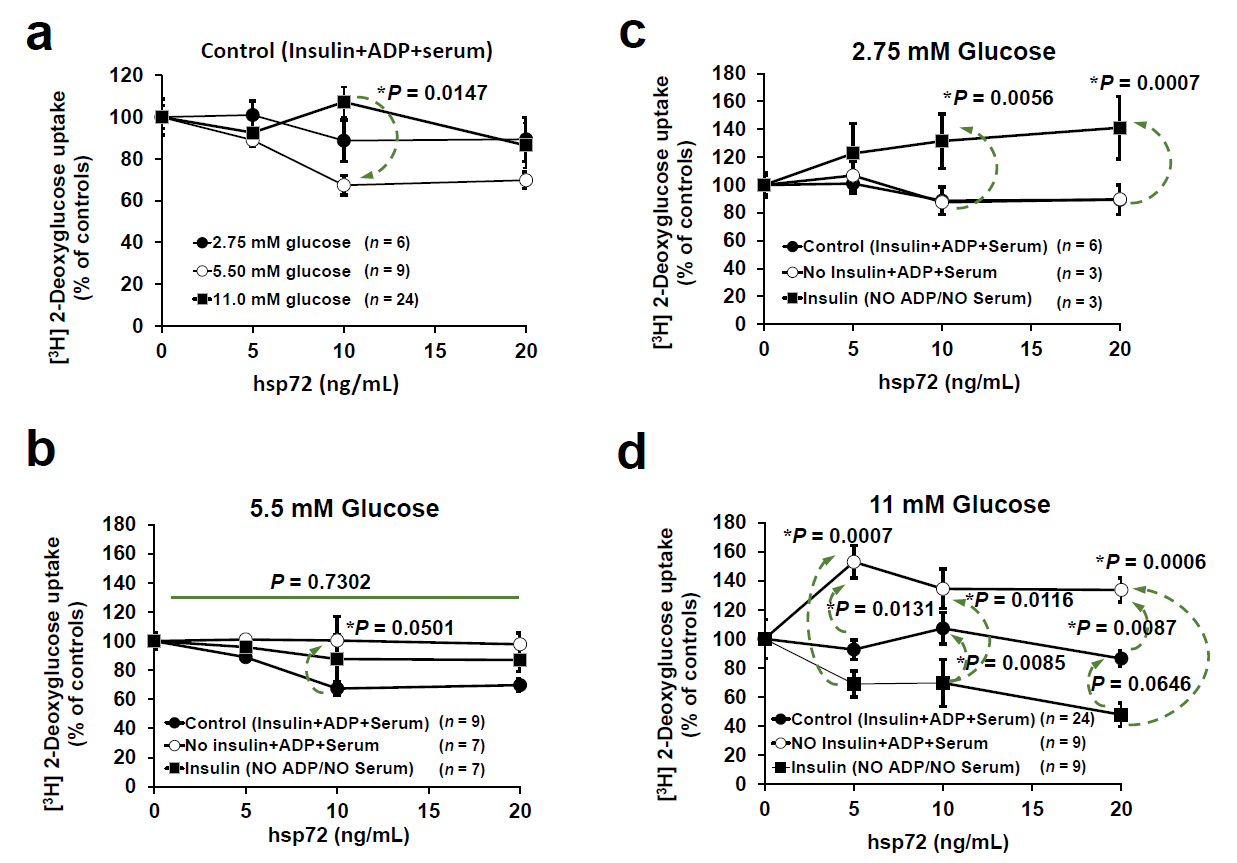


**Supplementary Fig. 9.** **HSP72 serum-dependently impairs [^3^H]-2DG uptake in soleus muscle incubated *ex vivo* with 5.5 mM glucose.** Twelve-hour fasted rats were killed and soleus muscles surgically excised and equilibrated for 30 min in modified Warburg flasks under agitation at 37 °C in HBSS gasified with 5 % CO_2_/95 % O_2_ (v/v) carbogen mixture, pH 7.4. After the equilibration phase, the muscle strips were incubated under agitation at 37 °C for additional 30 min in the presence or absence of 10 μU/mL insulin (60 pM, which is equimolar to 4.3 ng/mL HSP72) in HBBS containing (or not) dialysed autologous serum (we tested 0, 5 and 10 % by volume and chose 10 % in the subsequent experiments), 1 mM ADP (disodium salt) and increasing concentrations of recombinant HSP72 (rat/mouse LPS-free hspa1a at 0, 5, 10, 20, 100 or 500 ng/mL, which is equivalent to 0, 69.5, 139, 278, 1390 and 6950 pM, respectively) under the same above conditions. Because incubating tissues with concentrations of HSP72 higher than 20 ng/mL did not affect the results anymore, the panels illustrated only HSP72 additions up to 20 ng/mL. The experiments started with the addition of [^3^H]-2DG (0.47 μCi/mL) in either 2.75, 5.5 or 11 mM cold glucose. After incubations, samples were immediately freeze-clamped (liquid N_2_) and processed for liquid scintillation. **(a)** Control conditions (insulin + ADP + serum) and different glucose concentrations; **(b)** different conditions at 2.75 mM glucose; **(c)** different conditions at 5.5. mM glucose; **(d)** different conditions at 11 mM glucose. Data, expressed as the means ± s.d., were analysed by 2‑way RM ANOVA followed by Tukey’s multiple comparison testing. Sample size is shown in the figures.


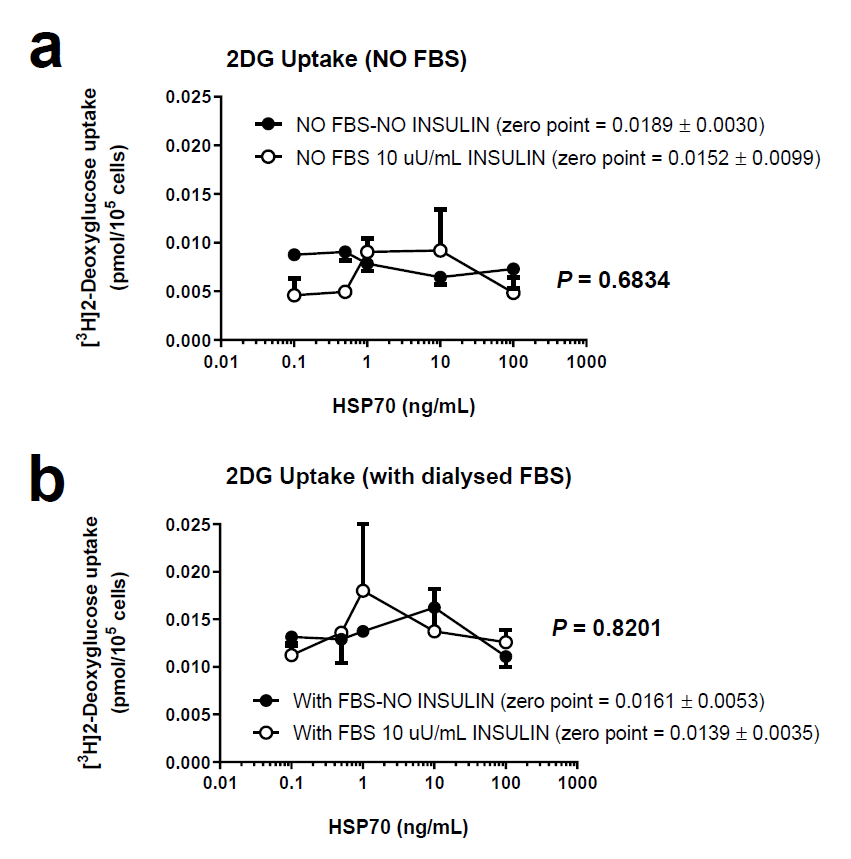
 **Supplementary Fig. 10. HSP72 does not alter [^3^H]-2DG uptake by L6 myoblasts in the presence of physiological concentrations of insulin *in vitro*.** Rat myoblast L6 cells were differentiated as described in the Methods section. In the morning of the experiment, medium was replaced with serum-free medium containing no glucose for 20 min, and then cells were exposed to increasing concentrations of recombinant rat/mouse LPS-free HSP72 (hspa1a at 0, 5, 10, 20, 100 or 500 ng/mL, which is equivalent to 0, 69.5, 139, 278, 1390 and 6950 pM, respectively) in RPMI 1640 medium containing 4.5 x 10^-3^ μCi/mL [^3^H]-2DG in 5.5 mM glucose, in the presence or absence of 10 μU/mL insulin as well as in the presence or absence of dialysed FBS (10 % by volume) for 15 min at 37 °C. We tested 0, 2, 5 and 10 % (v/v) dialysed FBS and 10 % FBS was found to account for the best concentration for glucose uptake by the cells. After the 15 min incubations, the dishes were placed on an ice bath and the cells washed in ice-cold PBS to be digested in 0.2 M NaOH for 1 h at 60 °C. Then, the samples were prepared to be counted by liquid scintillation. [^3^H]‑2DG uptake in the absence (a) or the presence (b) of dialysed FBS. Data, expressed as the means ± s.d., were analysed by 2‑way RM ANOVA followed by Tukey’s multiple comparison testing. Experiments were replicated twice with *n* = 2 for each test point.
